# Spread potential and architectural variant of a multiple-drug resistant *Escherichia coli* co-producing lipopolysaccharide-modifying determinants and extended-spectrum β-lactamases

**DOI:** 10.3389/fmicb.2026.1924788

**Published:** 2026-09-08

**Authors:** Shuang Wan, Tao Huang, Jun Li, Na Lv, Jinfeng Lu, Yanan Zhang, Aiping Zhou, Rongrong Chen

**Affiliations:** 1 College of Animal Science and Technology, Jiangxi Agricultural University, Nanchang, China; 2 College of Bioscience and Bioengineering, Jiangxi Agricultural University, Nanchang, China; 3 College of Food Science and Engineering, Jiangxi Agricultural University, Nanchang, China; 4 Department of Laboratory Medicine, Shanghai East Hospital, School of Medicine, Tongji University, Shanghai, China

**Keywords:** combinatorial antimicrobials, genomics and transcriptomics, horizontal transfer, lipopolysaccharide modification, multi-drug resistant *Escherichia coli*

## Abstract

**Background:**

Abuse of β-lactam antibiotics and its spread of drug resistance challenged the applicability of antimicrobial agents, enabling polymyxins as a last-line treatment for anti-infections of multi-drug resistant (MDR) pathogen. Polymyxin is applicable to herb breeding/growth prompting and combatting Gram-negative bacteria by combining other antimicrobials by membrane dysfunction, permeability damage, vesicle–vesicle contacts and oxidative stress, with its increasingly ubiquitous resistance. However, the complex mechanisms and high transmission risk of polymyxin resistance pose a severe threat to public health, such as improved colonization advantages and infection risks of *Escherichia coli*.

**Methods:**

In this study, a polymyxin-resistant *Escherichia coli* strain capable of producing extended-spectrum β-lactamase (ESBL), thereafter designated as EcE.ESBL.COL, was isolated from a urine sample. Antimicrobial susceptibility testing and whole-genome sequencing revealed that this strain likely harbors multiple antibiotic resistance determinants.

**Results:**

Therefore, these candidates were found on one chromosome [an integron-like MDR region not detected in another ST1485 representative strain (O83:H42)] and three plasmids (including *bla*_TEM − 1B_ and *bla*_CTX − M−55_) in this strain, pertaining to multi-locus sequence type (MLST) 1485 and serotype O25:H42. Interestingly, this genomic region of plasticity with similar arrangements located on either chromosome or plasmid in other distantly-related strains [for example, the chromosome of *E. coli* strain LWY24 (previously as ST93, reclassified into ST457 in our study) and plasmid pFXCREC004-1, harbored by *E. coli* strain FXCREC004 (ST101)]. Next, an *arnBCADTEF* operon and no *mcr*-like determinant was found on EcE.ESBL.COL chromosome, facilitating the 4-amino-4-deoxy-L-arabinose (L-Ara4N) modification of lipid A in lipopolysaccharide (LPS). Interestingly, polymyxin resistance conferred by *arn* was augmented by additional polysaccharides retrieved from *Lycium barbarum* (LBP) and antioxidant N-acetylcysteine (NAC), while no significant antimicrobial coordination was observed if combined with colistin. In addition, sodium dodecyl sulfate-polyacrylamide gel electrophoresis (SDS-PAGE) and comparative transcriptomics demonstrated that the LPS production and morphology significantly varied in response to polymyxin stress, indicating that polymyxin can induce the transcription of *arnBCADTEF*, thereby promoting L-Ara4N modification and polymyxin resistance.

**Conclusion:**

Overall, our finding indicates that the co-occurrence of antibiotic resistance factors (such as MDR candidates and LPS architectural variation in this isolate) involves both chromosomal recombination and plasmid dissemination, thereby highlighting important implications for antimicrobial resistance surveillance, molecular epidemiological retrospection, and precision clinical anti-infection.

## 1 Introduction

Currently, the misuse and overuse of antibiotics have accelerated the emergence and spread of multi-drug resistant (MDR) bacteria. In the context of their dissemination, polymyxins have become the last-line therapeutic option for anti-infections treatment upon extensively drug-resistant pathogens (Nang et al., 2021; Doijad et al., 2023). However, the diverse mechanisms and convergence of polymyxin and β-lactams resistance in bacteria constitute significant challenges to clinical anti-infection, public health and antimicrobial applicability, benefiting the colonization advantages contributing to various opportunistic hospital- and community-acquired diseases by multi-drug resistant *Escherichia coli* (Hammad et al., 2022; Abavisani et al., 2023; Dalmasso et al., 2023).

To our knowledge, polymyxins exert bactericidal activity by targeting lipopolysaccharide (LPS) in the outer membrane of Gram-negative bacteria, mainly by membrane disruption, anti-endotoxin, vesicle-vesicle contact, respiratory enzyme inhibition, oxidative damages driven by reactive oxygen species (Zhang et al., 2019; El-Sayed Ahmed et al., 2020). LPS consists of core polysaccharide, O-antigen, and lipid A, with lipid A serving as the primary binding site for polymyxins (Tang et al., 2025). Likewise, polymyxins resistance involves two-component system regulation, LPS structural modification, efflux pump activation, and altered membrane protein expression. Among these, LPS modification is a key mechanism by transferring phosphoethanolamine (pEtN) mediated by the plasmid-borne *mcr* (mobile colistin resistance), or L-Ara4N addition mediated by the chromosomal *arnBCADTEF* (Padhy et al., 2024, abbreviated: *arn* operon for short). These modifications related to lipopolysaccharides reconfiguration commonly occurred at distinct sites and reduce the negative charge on bacterial surface, in turn diminishing the electrostatics between polymyxin and LPS, challenging the clinical applicability of polymyxin (Breazeale et al., 2005; Olaitan et al., 2014). Currently, a variety of resensitization adjuvants (such as EDTA, polysaccharides and magnolol) were widely found to inhibit LPS modification or oxidative homeostasis, although the synergistic/augmented targets required to be further clarified (Cheng et al., 2024; Padhy et al., 2024; Diao et al., 2025; Zhai et al., 2025; Wu et al., 2026; Zhao et al., 2026).

In this study, an *Escherichia coli* strain designated as EcE.ESBL.COL was isolated from a clinical urine specimen, identified as ST1485 with serotype O25:H42. Antimicrobial susceptibility testing confirmed its resistance to polymyxin and a variety of β-lactams. Whole-genome sequencing revealed that the chromosome harbors an *arn* operon, which mediates LPS modification and confers polymyxin resistance. Notably, this resistance pattern was not found in other ST1485 representatives co-producing MCR and Arn (O83:H42). Interestingly, its polymyxin killing was attenuated by additional *Lycium barbarum* polysaccharides, and the dynamics of serial genes related to polymyxin resistance was investigated. Also, it is feasible that other resistance genes (such as *bla*_TEM − 1B_, *bla*_CTX − M−55_, *fosA3* and *dfrA14*) harbored by integrons and plasmids thereby maintain a trade-off between antibiotic resistance and growth/fitness costs. Interestingly, strain EcE.ESBL.COL retained the *arn* operon mediated polymyxin resistance. We speculated that this genetic organization likely contributed to decreased fitness cost induced by convergence of *arn* operon and *mcr*. Taken together, the underlying co-occurrence of antibiotic resistance genes may be diverse and complicate under milieu stress, and our findings would offer significant implications for targeted treatment strategies by using antibiotic adjuvants.

## 2 Materials and methods

### 2.1 Bacteria isolation and antibiotic susceptibility testing

The specimen was obtained from the urine of a hospitalized patient. After resuscitation and proliferation in suitable culture medium, it was inoculated in MacConkey agar for isolation and purification. After incubation at 36 ± 1 °C with for 18–24 h, the colonial morphology was observed. Then single colonies were analyzed using the VITEK 2 system (bioMérieux, Marcy-l'Étoile, France), with the AST-N335 for antibiotic susceptibility testing.

According to the guidelines of the clinical and laboratory standards institute (CLSI, Rai et al., 2023), antimicrobial susceptibility testing was performed for 29 antibiotics. Among them, the minimum inhibitory concentrations (MIC) of 18 antibiotics were determined using the broth microdilution method, while the inhibition zone diameters of the remaining 11 antibiotics were measured by the disk diffusion method. All assays (broth microdilution and disk diffusion) were performed in triplicate to ensure reproducibility, following CLSI M100 ED36 guidelines. *Lycium barbarum* polysaccharides (LBP) was purchased from Psaitong (Beijing, China), and N-acetylcysteine (NAC) was purchased from Macklin (Shanghai, China).

### 2.2 Growth curve

The strain was inoculated into Mueller-Hinton broth (MHB). Aliquots of the suspension were then distributed into the following groups in triplicate: a control group (without any treatment), colistin alone at 0.5 × MIC, colistin alone at 1 × MIC, colistin (1 × MIC) combined with LBP (2 mg/mL), and colistin (1 × MIC) combined with NAC (0.25 × MIC). All cultures were incubated at 37 °C for 24 h with continuous shaking. During the incubation, the optical density at 600 nm (OD_600_) was measured hourly.

### 2.3 Checkerboard assay and time-dependent killing curve

To investigate the change in antibacterial activity of colistin combined with LBP or NAC, the fractional inhibitory concentration index (FICI) was determined using the checkerboard method (Cheng et al., 2024). First, 50 μL of different concentrations of colistin were added horizontally in a 96-well plate, and an 8 × 8 matrix was prepared followed by 50 μL of different concentrations of LBP or NAC added vertically. Then, 100 μL of bacterial suspension was added to each well and mixed. After incubation at 37 °C for 16–18 h, the optical density of each well was measured at 600 nm (OD_600_). The combined effect of the drug pair was quantified by the Bliss score using the R package synergyfinder (v3.16.0), with δ < −10 for antagonism, −10 ≤ δ ≤ 10 for additivity or inconclusive interaction, and δ > 10 for meaningful synergy, respectively (Zheng et al., 2022; Petakh et al., 2026). The FICI was calculated using the following formula: FICI = FIC_a_ + FIC_b_ = MIC_ab_ / MIC_a_ + MIC_ba_ / MIC_b_. Moreover, FICI ≤ 0.5 indicates a synergistic effect between drugs a and b; 0.5 < FICI ≤ 4.0 indicates no significant coordination; FICI > 4.0 indicates an antagonistic effect between selected two drugs (Odds, 2003).

After serial dilution of EcE.ESBL.COL culture in the logarithmic phase, a control group (without any drug), a colistin (1/2 × MIC) group, a colistin (1 × MIC) group, a colistin (1 × MIC) + LBP (2 mg/mL) group, and a colistin (1 × MIC) + NAC (1/4 × MIC) group were prepared, respectively. Next, these combinatorial mixtures were incubated in a shaker at 37 °C and 380 rpm for 24 h, and samples were acquired every 2 h for plate colony counting in triplicate.

### 2.4 Conjugation experiments

To confirm whether the resistance genes of the strain EcE.ESBL.COL can be horizontally transferred to other bacteria, *Escherichia coli* 25DN was available as the recipient in this conjugation assay (Fu et al., 2020). Briefly, the strain EcE.ESBL.COL and 25DN were mixed at a 1:3 ratio, and the mixture was spotted onto a membrane filter placed on Luria-Bertani (LB) agar, followed by incubation at 37 °C for 18–24 h. The conjugates on the filter were scratched off with 1 mL LB broth, and the suspension was diluted and spread onto LB plates containing 6 μg/mL ampicillin alone or 6 μg/mL ampicillin plus 2 μg/mL sodium azide. After incubation at 37 °C for 20–24 h, transformant colonies growing on the plates with both selective agents were picked and subjected to PCR verification.

### 2.5 Detection of reactive oxygen species

The reactive oxygen species (ROS) levels of EcE.ESBL.COL after treatment with serial concentrations of colistin (0, 1/4 × MIC, 1/2 × MIC, 1 × MIC, 2 × MIC), LBP (0, 0.25, 1, 4 mg/mL), and NAC (0, 1/16 × MIC, 1/4 × MIC, 1 × MIC) in combination were detected using the 2′7′-Dichlorodihydrofluorescein diacetate (DCFH-DA) fluorescent probe (UElandy, Suzhou, China). Briefly, an appropriate amount of log-phase bacterial culture was centrifuged and resuspended in an equal volume of phosphate buffered solution (PBS) containing 100 μM DCFH-DA. After incubating at 37 °C for 1 h, the mixture was washed once with PBS. Then, the bacterial suspension was mixed with the corresponding concentration of drugs and incubated at 37 °C for 4 h. Fluorescence values were measured at an excitation wavelength of 488 nm and an emission wavelength of 525 nm (Wei et al., 2018).

### 2.6 SDS-PAGE and limulus amebocyte lysate assay of LPS

LPS was extracted from cultures of strain EcE.ESBL.COL, co-cultured with polymyxin, control strain ATCC 25922, and an LPS-deficient mutant to evaluate the effect of polymyxin on LPS production or modification. LPS extraction was performed using a Lipopolysaccharide Extraction Kit (Solarbio, Beijing, China) according to the following protocol: bacterial cells were collected by centrifugation at room temperature, resuspended in 500 μL of Reagent A, and centrifuged at 8,000 × g for 3 min, after which the supernatant was discarded. The pellet was collected, resuspended in 500 μL of Reagent A, and centrifuged again at 8,000 × g for 3 min, followed by removal of the supernatant. The resulting pellet was resuspended in 500 μL of Extraction Buffer B and centrifuged at 10,000 × g for 10 min; the supernatant was discarded. To the supernatant collected from the previous step, 500 μL of Extraction Buffer C was added, and the mixture was vigorously vortexed for 20 s to ensure thorough mixing. The sample was incubated at 66–68 °C for 30 min, with gentle pipetting every 10 min to homogenize the mixture. After overnight incubation at 4 °C, the sample was centrifuged at 3,000 × g for 20 min at 4 °C, yielding the purified LPS.

Subsequently, SDS-PAGE was performed for LPS profiling of strain EcE.E.C using a discontinuous gel system consisting of a 12% separating gel and a 5% stacking gel. LPS samples were analyzed by SDS-PAGE in Tris-glycine-SDS running buffer at 110 V for 90 min. Subsequently, the LPS bands were visualized using a PAGE Gel Fast Silver Staining Kit (Solarbio, Beijing, China). The gel map was photographed by using QuickGel 6200 (Monad Biotech Co. Ltd., Suzhou, China).

The free lipopolysaccharide content in the samples was measured using a Gram-negative bacterial lipopolysaccharide detection kit (*Limulus amebocyte* lysate assay, Genobio Pharmaceutical Co. Ltd., Tianjin, China). 10 μL of the bacterial suspension to be tested was mixed with 40 μL of treatment solution (equal volumes of treatment solution A and treatment solution B), and treated at 37 °C for 10 min. Then, 200 μL of the reaction master solution was added and shaken for 1–2 s. The absorbance was measured at 405 nm, with three replicates for each group.

### 2.7 RT-qPCR

To investigate the effects of colistin combined with LBP and NAC on the lipopolysaccharide-related genes of EcE.E.C, we performed RT-qPCR analysis on the control group, the colistin (1/4 × MIC) group, the colistin (1/4 × MIC) + LBP (2 mg/mL) group, and the colistin (1/4 × MIC) + NAC (1/4 × MIC) group. After extracting total RNA from the samples using the Bacteria RNA Extraction Kit (Vazyme, Nanjing, China), RNA was reverse-transcribed into cDNA using FastSensi gDNA dispelling RT SuperMix (TIANGEN, Beijing, China). RT-qPCR analysis was performed using 2 × Universal SYBR qPCR Master Mix (GenOrigin, Beijing, China) on Archimed-X4 (Rocgene, Beijing, China, of note, gene primers designed by Primer Premier 5.0 (Supplementary Tables S1, S2). 16S rRNA was used as the internal reference gene. Of note, C_t_ mean ± standard deviation (SD), the coefficient of variation (CV)%: colistin group, 13.37 ± 0.20, 1.48%; colistin + LBP, 13.19 ± 0.14, 1.07%; colistin + NAC, 13.39 ± 0.43, 3.23% (fairly low variability with SD < 1, Chen et al., 2024), and the relative expression levels of the target genes were calculated using the 2^−Δ*ΔCt*^ method (Zhang et al., 2026).

### 2.8 Genomic sequencing and comparative transcriptomics

Genomic DNA was extracted using the Gentra Puregene Yeast/Bact. Kit (Qiagen, Valencia, CA). Sequencing was performed on the Illumina high-throughput sequencer and the GridION X5 system (Oxford Nanopore, Oxford, UK) by Zhejiang Tianke (Hangzhou, China) for EcE.E.C sequencing (raw reads: 1,47,283; N50: 21,124 bp; clean reads: 1,39,372; completeness: 93.92%). After filtering the raw sequencing read, Unicycler (v0.4.5, Wick et al., 2017) was employed for genome assembly and evaluation. The resulting genome assembly consisted of one chromosome and three plasmids, which were further annotated using a prokaryotic annotation pipeline. In addition, protein function was manually annotated by comparing protein sequences with the Non-Redundant Protein Sequence Database (NR, ftp.ncbi.nih.gov/blast/db), Swiss-Prot (www.ebi.ac.uk/uniprot), KEGG (www.genome.jp/kegg) and COG (*E*-value < 0.00001, www.ncbi.nlm.nih.gov/COG) databases.

Comparative transcriptomics were customized as follows: the colistin (1/4 × MIC) group, the colistin (1/4 × MIC) + LBP (2 mg/mL) group, and the colistin (1/4 × MIC) + NAC (1/4 × MIC) group. Strain EcE.ESBL.COL in these groups were cultured at 37 °C and 220 rpm until the logarithmic growth phase (three replicates). After treatment with liquid nitrogen, the samples were stored at −80 °C for RNA extraction and transcriptome sequencing: total RNA was extracted from the samples using the Trizol method, ribosomal RNA was removed using the Epicenter Ribo-Zero rRNA Removal Kit, and a strand-specific library was constructed using the NEBNext^®^ Ultra II™ Directional RNA Library Prep Kit for Illumina. The constructed libraries were sequenced using the Illumina high-throughput sequencing platform with PE150 sequencing, ultimately obtaining raw sequencing reads (RawReads).

### 2.9 Bioinformatics

Multilocus sequence typing (MLST) was performed on their housekeeping genes using MLST 2.0 (cge.food.dtu.dk/services/MLST). The housekeeping genes of each strain were concatenated in the order of *adk, fumC, gyrB, icd, mdh, purA*, and *recA*. Subsequently, the sequences of the selected strains were aligned and constructed using the neighbor-joining method with 1,000 bootstrap replicates by MEGA 11 (www.megasoftware.net). And the phylogeny was visualized with interactive tree of life (iTOL, itol.embl.de). The amino acid sequences of the EcE.ESBL.COL were compared against the virulence factor database (VFDB, www.mgc.ac.cn/VFs) to identify virulence factors. Concurrently, antibiotic resistance genes and serotypes were determined by using ResFinder 4.0 (cge.cbs.dtu.dk/services/ResFinder) and SerotypeFinder (cge.food.dtu.dk/services/SerotypeFinder), respectively. Genomic architectures, functional elements, and gene annotation information were compared through Proksee (proksee.ca) analysis. Blast Ring Image Generator (BRIG, sourceforge.net/projects/brig) was used to generate circular comparison for *E. coli* genomes. PlasmidFinder (cge.food.dtu.dk/services/PlasmidFinder) was employed to identify plasmid replicon genes and their corresponding incompatibility types.

Quality control of RNA-Seq RawReads was performed using fastp (v0.23.2, Chen et al., 2018) to remove sequencing adapters and low-quality sequences. The cleaned reads were aligned to the reference genome using Bowtie2 (v2.4.5, Langmead and Salzberg, 2012; Langmead et al., 2019). Transcript quantification was carried out using salmon (v1.9.0, Patro et al., 2017) and the R package tximport (Soneson et al., 2015). Differential gene expression analysis was performed using DESeq2 (v1.48.1, Love et al., 2014). Functional annotation of differentially expressed genes (DEGs) was conducted using eggnog-mapper (v5.0, Huerta-Cepas et al., 2019; Buchfink et al., 2021; Cantalapiedra et al., 2021). GO and KEGG pathway enrichment analysis of DEGs were performed using the R package clusterProfiler (v4.16.0, Wu et al., 2021).

### 2.10 Statistical analysis

Unless stated otherwise, all data were analyzed using R package rstatix (v0.7.3, rpkgs.datanovia.com/rstatix), and the results are presented as mean ± stand error (SE). The significance of differences between groups was assessed by using one-way ANOVA with Tukey's *post-hoc* test (^*^P < 0.05, ^**^P < 0.01, ^***^P < 0.001, ^****^P < 0.0001).

## 3 Results

### 3.1 Strain identification and antimicrobial susceptibility testing

This patient (information was anonymized, exemption from informed consent, Approval No. 2025YS-189, Ethics Committee of Shanghai East Hospital) was admitted to the hospital, obtained a urine sample. And then the sample was cultured on selective medium MacConkey agar at 36 ± 1 °C with for 18–24 h. Colony morphology is in agreement with the featural indicators of *Escherichia coli*.

The antimicrobial susceptibility testing was further conducted by using the VITEK 2 COMPACT system with the AST-N335. In addition, antimicrobial susceptibility testing showed that the strain is a MDR isolate, demonstrating antibiotic resistance to a total of 12 antimicrobial agents (Table 1). Interestingly, this isolate was identified to be resistant to various β-lactam antibiotics, including ampicillin, aztreonam, and cefuroxime. This profile is consistent with an extended-spectrum β-lactamase (ESBL) phenotype (interpreted by CLSI). The strain isolated from Shanghai East Hospital was also demonstrated to be resistant to colistin, with the MIC value of 16 μg/mL. Therefore, the strain is named EcE.ESBL.COL in this study.

**Table 1:** Antibiotic susceptibility testing for strain EcE.ESBL.COL.

Antimicrobial Type	Antibiotic	Abbr.^a^	MIC^b^ (μg/mL)	Zone d^c^ (mm)	R/S/I^d^
β-lactams	Ampicillin	AMP	–	6	R
	Ampicillin/Sulbactam	SAM	–	15	S
	Aztreonam	ATM	16	–	R
	Cefuroxime	CXM	–	6	R
	Piperacillin/Tazobactam	TZP	≤ 4	–	S
	Ticarcillin/Clavulanic Acid	TIM	16	–	S
Cephalosporins	Cefazolin	CZO	–	6	R
	Cefoperazone/Sulbactam	SCF	≤ 8	–	S
	Ceftriaxone	CRO	–	6	R
	Cefotaxime	CTX	–	6	R
	Ceftazidime	CAZ	8	–	I
	Cefepime	FEP	16	–	R
	Cefoxitin	FOX	–	22	S
Carbapenems	Ertapenem	ETP	–	30	S
	Meropenem	MEM	≤ 0.25	–	S
	Imipenem	IPM	≤ 0.25	–	S
Aminoglycosides	Gentamicin	GEN	–	18	S
	Amikacin	AMK	≤ 2	–	S
	Tobramycin	TOB	≤ 1	–	S
Polymyxins	Polymyxin B	PB	32	–	R
	Colistin	CST	≥16	–	R
Quinolones	Ciprofloxacin	CIP	1	–	R
	Levofloxacin	LVX	1	–	I
Tetracyclines	Doxycycline	DOX	≥16	–	R
	Minocycline	MIN	2	–	S
	Tigecycline	TGC	≤ 0.5	–	S
Fosfomycin	Fosfomycin	FOS	–	13	I
Nitrofurans	Nitrofurantoin	NIT/F	–	20	S
Sulfonamides	Trimethoprim/Sulfamethoxazole	–	≥320	–	R

^a^Abbr., abbreviation.

^b^The minimum inhibitory concentrations examined using VITEK 2 Compact AST-N335 with broth micro-dilution.

^c^d, diameter; determined by the Kirby-Bauer disc on MHA plates.

^d^R, resistant; S, susceptible; I, intermediate. Antimicrobial susceptibility testing results were interpreted according to the guidelines recommended by the clinical and laboratory standards institute (CLSI) with *E. coli* ATCC 25922 as the reference strain. In addition, inhibition zones on Müeller-Hinton agar were measured by Kirby-Bauer disc diffusion assays. Of note, 18 and 11 antimicrobials were applicable to micro-dilution and disk diffusion, whereas “-” denotes no corresponding testing.

**Figure 1:**
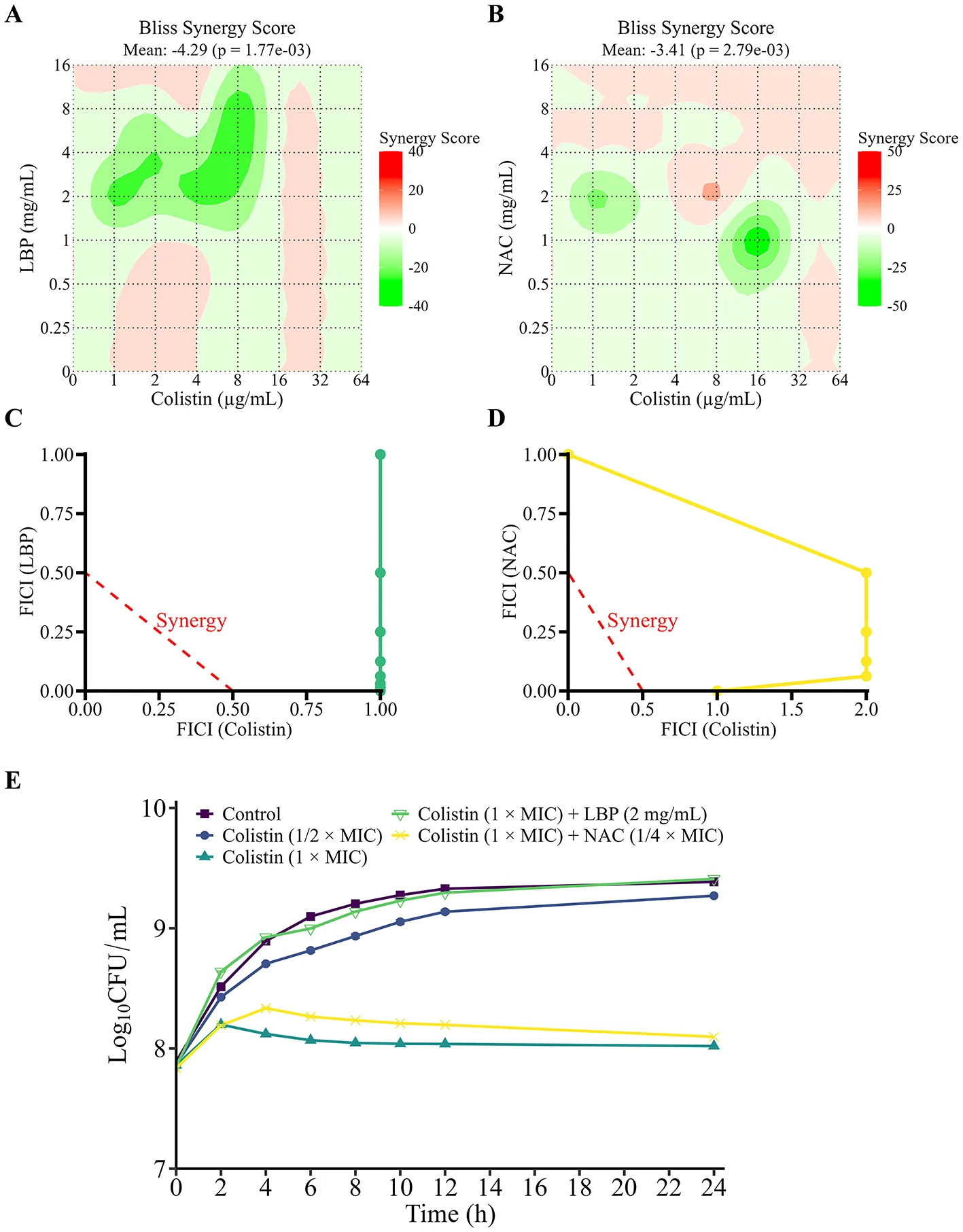
Checkerboard assay and time-dependent killing. **(A)** Antibacterial activity of colistin combined with LBP. **(B)** Antibacterial activity of colistin combined with NAC. Color intensity corresponds to the absorbance at 600 nm (three independent repeats). The red area indicates a synergistic response, while the green area indicates additive or antagonistic. **(C)** Isobolograms for the combinatorial treatment of colistin and LBP against the strain EcE.ESBL.COL, with FICI ranging from 1 to 2. **(D)** Isobolograms for the combinatorial treatment of colistin and NAC against the strain EcE.ESBL.COL, with FICI ranging from 2.0625 to 2.5. **(E)** Time-dependent killing curve. EcE.ESBL.COL was cultured under different treatment conditions at 37 °C with shaking for 24 h. At 2 h intervals, 50 μL of the bacterial suspension was sampled, diluted, and spread on plates for CFU counting. The corresponding results were presented as mean ± SE.

Of note, the MIC of NAC was 4 mg/mL and the MIC of LBP was >16 mg/mL. Interestingly, no significant antimicrobial activity was observed by additional LBP. In addition, FICI was not acquired throughout serial working concentrations of LBP by combining with colistin. As a result, we selected 16 mg/mL as the highest content of LBP according NAC concentrations. Checkerboard assay showed that the FICI was ranging from 1 to 2 if combined with LBP, indicating no significant coordination effects, while NAC also had no significant effect on the MIC of colistin (Figures 1A, C). The FICI of NAC combined with colistin ranged from 2.0625 to 2.5. When 1 mg/mL of NAC was added, the MIC of colistin increased from 16 μg/mL to 32 μg/mL (Figures 1B, D). These findings prompted us to investigate the combinatorial bactericidal effect of colistin with LBP and NAC, we thus compared the killing curves of EcE.ESBL.COL under the treatment of colistin alone and in combination with LBP and NAC. If 16 μg/mL of colistin added into the mixture, both LBP and NAC could attenuate the bactericidal effect of colistin. Especially, LBP significantly reduced the bactericidal effect of colistin (Figure 1E). Interestingly, fitness cost upon bacterial growth driven by additional polysaccharides LBP and antioxidant NAC was observed, respectively (Supplementary Figure S1). This finding showed fitness cost associated with LBP/NAC combination treatment. Next, we assessed that the occurrence and dynamics of *mcr-* or *arnT-*like LPS-modifying determinants in this strain could likely monitor the trade-off between bacterial growth and fitness cost (Zhang et al., 2019; Lu et al., 2022). Consequently, we attempted to investigate the genomics and transcriptomics of this strain to reveal the likely dynamics of lipid A modifying determinants under LBP or NAC treatment.

### 3.2 Genome sequencing and general characteristics of EcE.ESBL.COL

Whole genome sequencing revealed that the strain EcE.ESBL.COL harbors a chromosome of approximately 5.04 Mb with GC content of 50.46%, and three plasmids. The plasmids measure 167,587 bp, 78,689 bp, and 1,998 bp in size, with corresponding GC contents of 50.41%, 52.99%, and 40.89%, respectively. Of note, numerous genes responding to the observed antibiotic resistance were identified, such as sulfonamides (*sul2*), aminoglycosides [*(aph3*′*)-Ib* and *aph(6)-Id*], tetracyclines [*tet(A)*], and amphenicols (*floR*, Supplementary Table S3). To further investigate the genetic characteristics and transmission mechanisms of these genes, we conducted a comparative analysis to investigate their sequence types and the genetic diversity of antimicrobial resistance dissemination.

### 3.3 Comprehensive genomics of EcE. E.C

**Figure 2:**
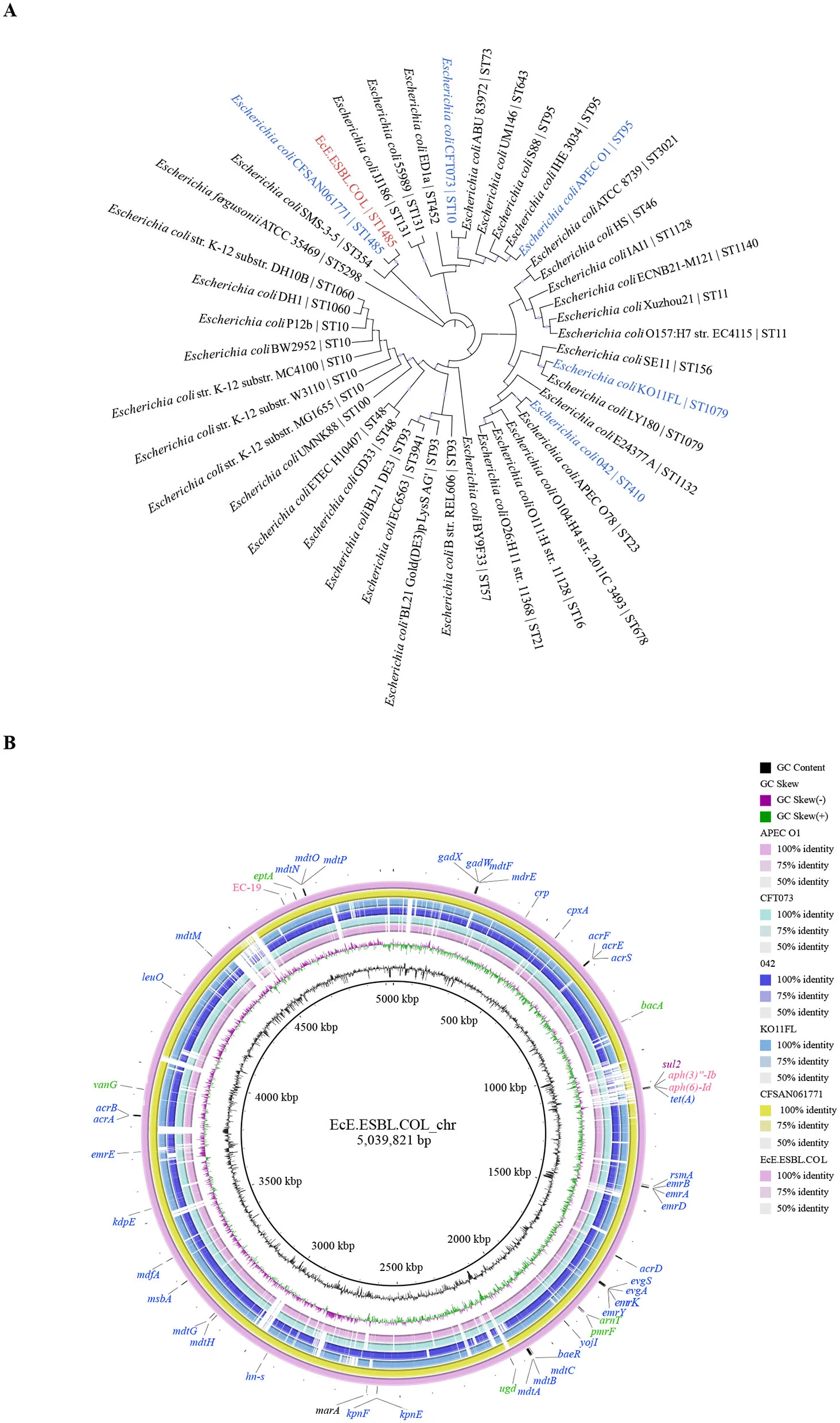
Phylogenetic analysis and circular diagram of strain EcE.ESBL.COL chromosome. **(A)** Phylogenetic analysis of strain EcE.ESBL.COL and *E. col*i strains with different sequence types. Based on MLST typing resulting from seven housekeeping genes, the phylogeny was constructed using the neighbor-joining method with 1000 bootstrap replicates by MEGA 11 (www.megasoftware.net), and was subsequently visualized and optimized using the Interactive Tree of Life (iTOL, itol.embl.de). Red: the isolated strain in this study. **(B)** Circular diagram of strain EcE.ESBL.COL chromosome. From the innermost to the outermost ring: GC content, GC skew, strains of 042, APEC O1, CFT073, 042, KO11FL, CFSAN061771, and EcE.ESBL.COL. Antibiotic resistance genes were predicted using the Resistance Gene Identifier (RGI) from the Comprehensive Antibiotic Resistance Database (CARD). And schematic diagram was constructed with the Blast Ring Image Generator (BRIG). The color scheme denotes resistance mechanisms: blue (antibiotic efflux), pink (antibiotic inactivation), green (antibiotic target alteration), purple (antibiotic target modification), and black (combined antibiotic efflux and reduced permeability to antibiotics).

We revealed that strain EcE.ESBL.COL shares an identical sequence type (ST1485) with strain CFSAN061771, so we selected other 41 strains with different sequence types to construct a phylogenetic tree (Figure 2A). Consequently, representative *E. coli* strains (such as strains CFSAN061771, KO11FL, 042, CFT073, and APEC O1) were subjected to following analysis for general genomic information (Figure 2B), virulence factors and antibiotic resistance.

Interestingly, strains EcE.ESBL.COL and CFSAN061771 share the same H-antigen, but differ in their O-antigen by analyzing their serotypes and O-antigen genes. In short, strain EcE.ESBL.COL was O25, while strain CFSAN061771 was O83 (Supplementary Table S4). Further genomic analysis revealed the genome of strain EcE.ESBL.COL carries a gene encoding UDP-glucose 4-epimerase with different coding functions (Table 2). This enzyme catalyzes the conversion of glucose to galactose in the O-antigen biosynthesis pathway, leading to structural variations in O-antigen (Roper and Ferguson, 2003; Nagode et al., 2023). Genomic analysis of strain CFSAN061771 revealed two key enzymes involved in O83-family O-antigen synthesis: O-antigen flippase and O-antigen polymerase (Supplementary Table S5). The emergence and variation of the genes encoding these enzymes are likely the main attributes of the structural differences in O-antigen observed in strain CFSAN061771.

**Table 2:** Comparison of O-antigen related genomic information between *E. coli* strain EcE.ESBL.COL and strain CFSAN061771.

Locus tag	Length (bp)	Gene	Product
*E. coli* strain EcE.ESBL.COL (O25:H42)			
ACYG3R_09925	344	*galE*	UDP-glucose 4-epimerase
ACYG3R_09930	368	*wbjC*	UDP-2-acetamido-2,6-beta-L-arabino-hexul-4-ose reductase
ACYG3R_09935	376	*wecB*	non-hydrolyzing UDP-N-acetylglucosamine 2-epimerase
ACYG3R_09940	402	*gtr4*	glycosyltransferase family 4 protein
ACYG3R_09945	135	*wbuC*	WbuC family cupin fold metalloprotein
ACYG3R_09950	468	*gndA*	6-phosphogluconate dehydrogenase
ACYG3R_09955	388	*ugd*	UDP-glucose 6-dehydrogenase
*E. coli* strain CFSAN061771 (O83:H42)			
CA902_008765	297	*galF*	UTP–glucose-1-phosphate uridylyltransferase GalF
CA902_008770	480	*wzx*	O83 family O-antigen flippase
CA902_008775	411	*wzy*	O83 family O-antigen polymerase
CA902_008780	301	*gtr2*	glycosyltransferase family 2 protein
CA902_008785	239	-	glycosyltransferase
CA902_008790	181	-	hypothetical protein
CA902_008795	236	-	WecB/TagA/CpsF family glycosyltransferase
CA902_008800	273	-	LysR family transcriptional regulator
CA902_008805	468	*gndA*	NADP-dependent phosphogluconate dehydrogenase
CA902_008810	388	*ugd*	UDP-glucose 6-dehydrogenase

Also, VFDB-driven analysis also revealed differences in virulence-related genes (Supplementary Tables S5, S6). For example, regarding *P* fimbriae, strain EcE.ESBL.COL carries *papB* and *papI* whereas strain CFSAN061771 only harbors *papX*. Additionally, strain EcE.ESBL.COL contains the iron-regulated element IreA and capsule (acinetobacter). However, EcE.E.C lacks GalE (present in strain CFSAN061771), associated with exopolysaccharide synthesis.

**Figure 3:**
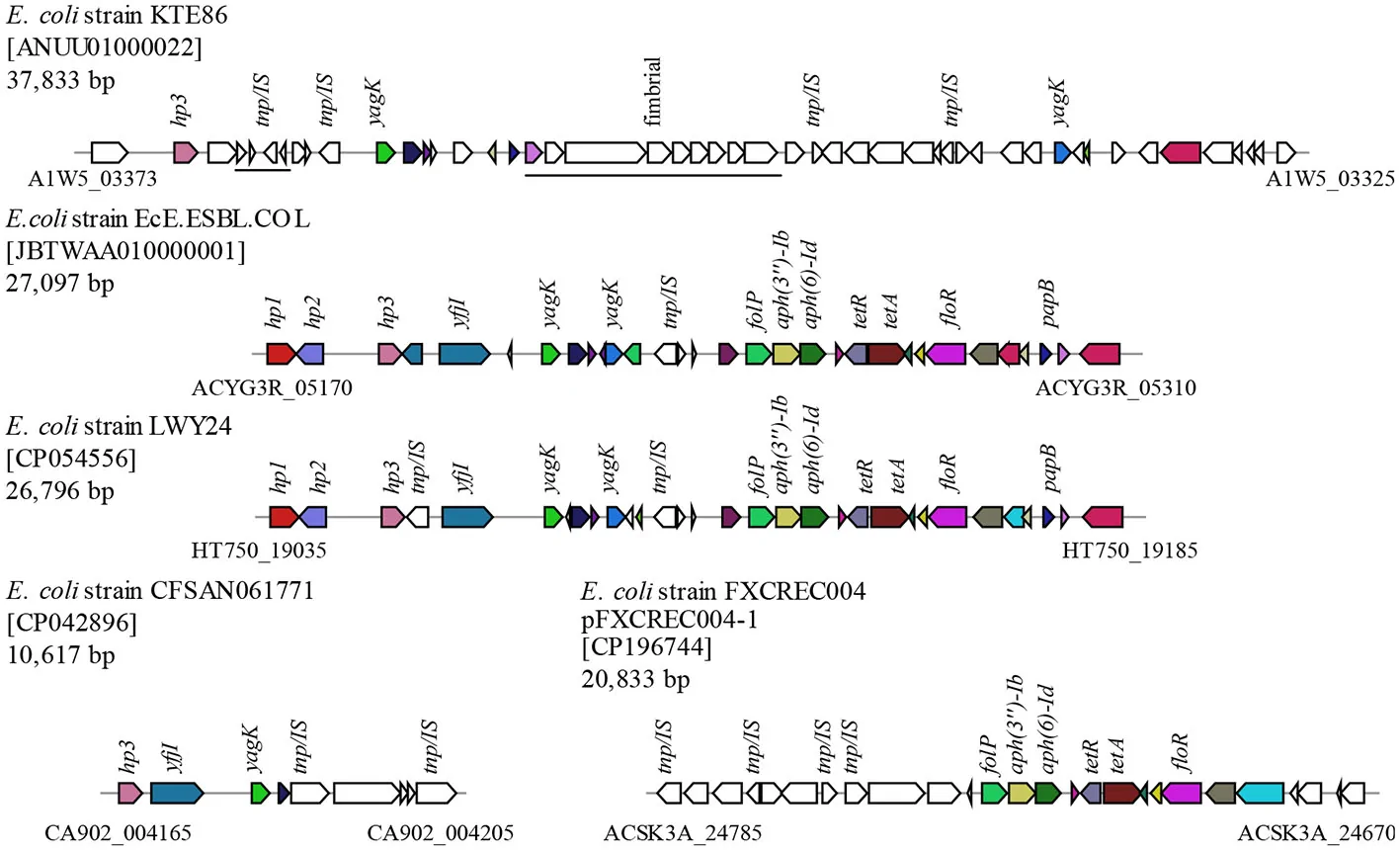
Genetic context comparison of the antimicrobial resistance genes. The sequences aligned (from top to bottom) are as follows: the EcE.ESBL.COL chromosome (this study), the LWY24 chromosome, plasmid pFXCREC004-1, ST1485 strain CFSAN061771 chromosome, and the KTE86 chromosome, aimed to investigate the transmission characteristics of this resistance gene. Note: *hp*, the gene encoding a hypothetical protein. The schematic diagram was generated by multigeneblast (accessible at multigeneblast.sourceforge.net), while the same color indicates homologous genes.

Genomic analysis revealed that serial MDR genes in EcE.ESBL.COL are located within a genomic variable region of approximately 5.2 kbp, harboring resistance genes for sulfonamides (*sul2*), aminoglycosides *(aph3*″*)-Ib* and *aph(6)-Id*, tetracyclines [*tet(A)*], and amphenicols (*floR*, Figure 3). Notably, the intact resistance region was absent in strain CFSAN061771, but its homologs or remnants were found in the chromosomes or plasmids of other strains. For example, the presence of this MDR region-like derivatives/variations were observed on the chromosome of *E. coli* strain LWY24 (previously as ST93, reclassified into ST457) and IncFII-IncX3 hybrid plasmid pFXCREC004-1 harbored by *E. coli* strain FXCREC004 (ST101).

**Figure 4:**
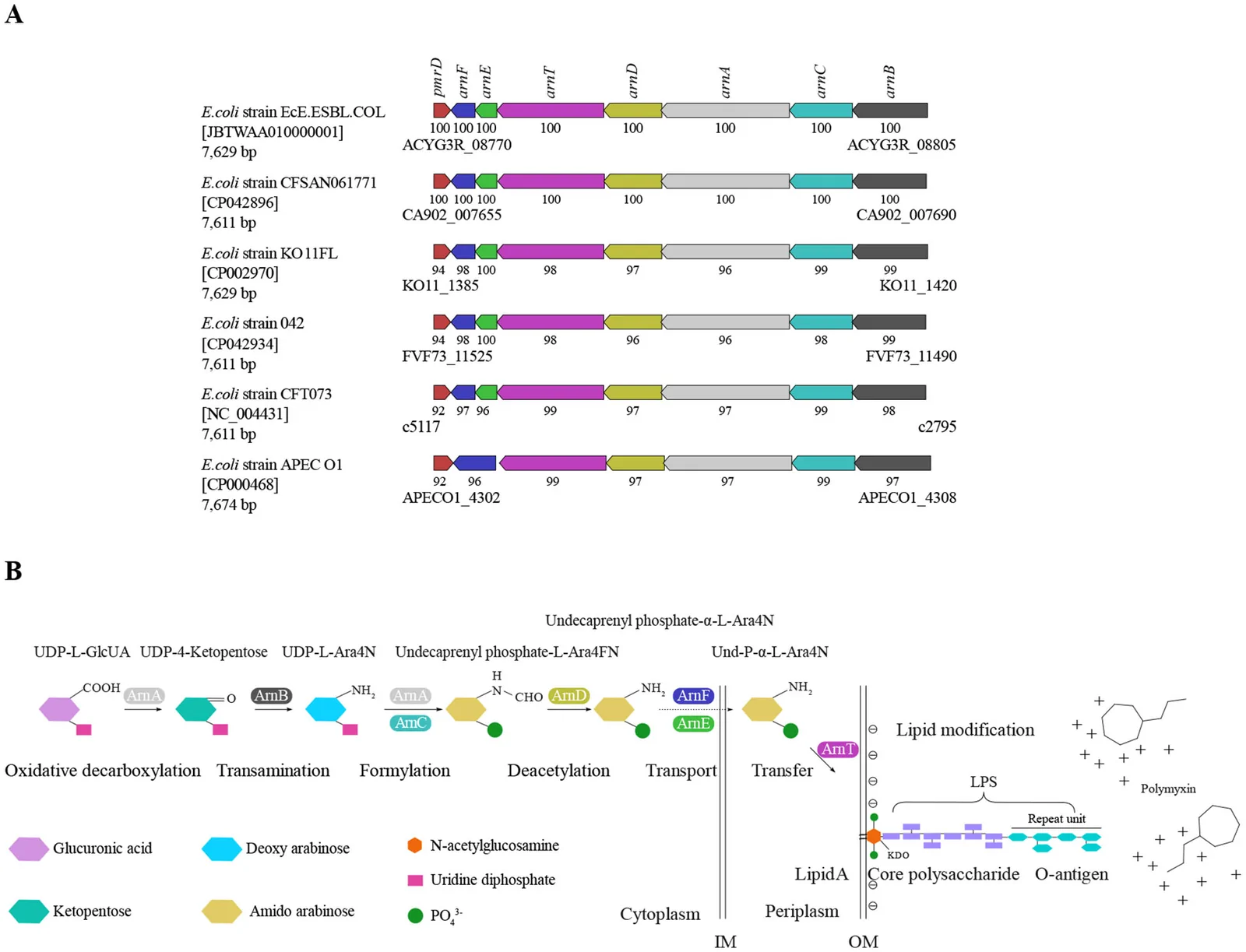
Comparative analysis of the *arnBCADTEF* similarity among different strains and the biosynthetic pathway of L-Ara4N modification. **(A)** The co-linearity comparison of the *arnBCATDEF* cluster in strains EcE.ESBL.COL, CFSAN061771, KO11FL, 042, CFT073 and APEC O1. These comparative schematics were calculated by multigeneblast and drawn to scale. Note: Numbers denote sequence identity (100 = 100%) against the reference against our genome. **(B)** Schematics of the L-Ara4N modification pathway. LPS, lipopolysaccharide; IM, inner membrane; OM, outer membrane.

This comparison suggested that antimicrobial resistance genes can disseminate via horizontal gene transfer, resulting in remarkable divergence in adaptability characteristics among closely-related strains (such as isolates with the same STs, Ellabaan et al., 2021). In addition, an intrinsic *arnBCADTEF* operon likely accounting for lipopolysaccharide modification and resulting polymyxin resistance was also found in EcE.ESBL.COL (Figure 4 and Supplementary Table S7). Thus, this study provides an insight into the co-occurrence dynamics of antimicrobial resistance genes.

### 3.4 Plasmid replicon types and featural genes

Plasmid replicons were typed by PlasmidFinder 2.0. Both pEcE.ESBL.COL-01 and pEcE.ESBL.COL-02 are IncF type plasmids. Plasmid replicons IncFIA, IncFIB (AP001918), and IncFIC (FII) were detected in pEcE.ESBL.COL-01, whereas IncFII (pHN7A8) was detected in pEcE.ESBL.COL-02 (virulence factors shown in Supplementary Table S6).

**Figure 5:**
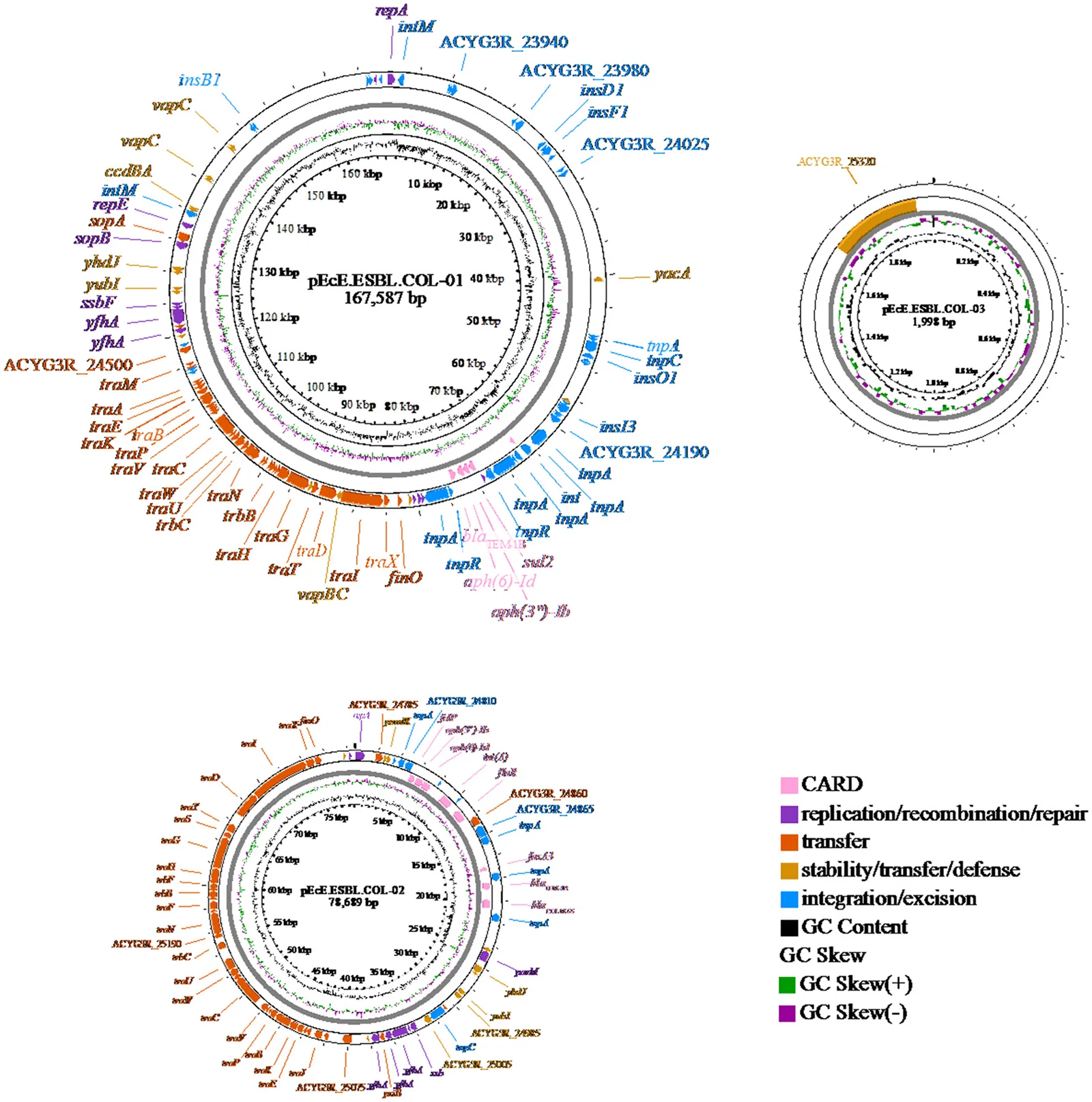
Circular diagrams of strain EcE.ESBL.COL plasmids. **(A)** pEcE.ESBL.COL-01, **(B)** pEcE.ESBL.COL-02 and **(C)** pEcE.ESBL.COL-03. From inner to outer layers: GC content, GC skew, and mobile genetic element analysis. GC Skew was predicted using BRIG analysis, antimicrobial resistance genes were assessed by ResFinder, and mobile genetic elements were identified via Proksee.

**Figure 6:**
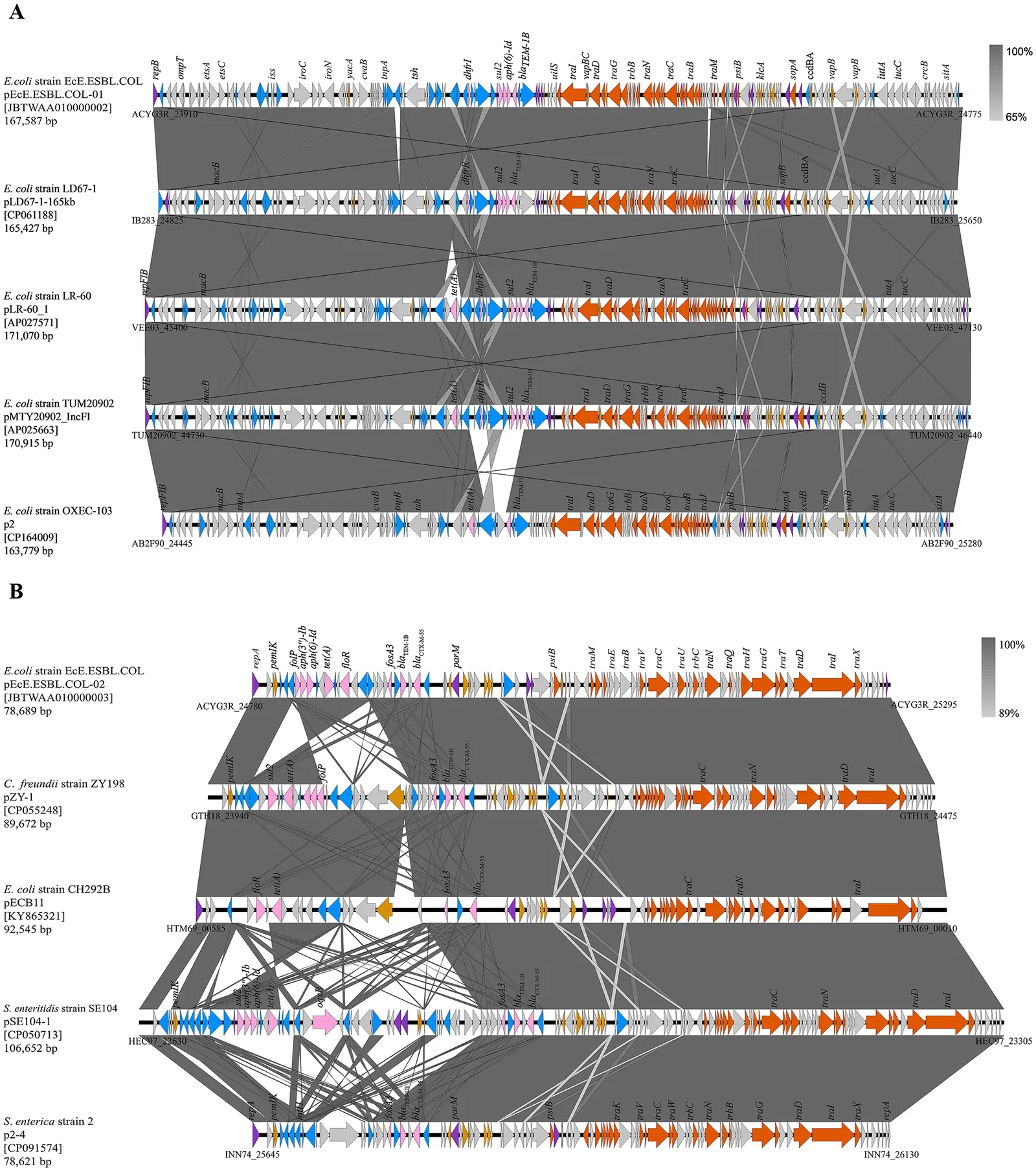
Linear alignment for two plasmids and their homologs. Synteny of **(A)** pEcE.ESBL.COL-01, **(B)** pEcE.ESBL.COL-02 and their homologous plasmids were generated by Easyfig. Homologous regions are marked by gray shading. The transcription directions of genes are indicated by arrows. Resistance genes, conjugal transfer genes, replication/recombination/repair genes, stability/transfer/defense genes and integration/excision genes are shown in pink, orange, purple, yellow and blue, respectively. Other genes are shown in gray.

Next, the spread of plasmid mediated resistance genes is an important pathway for bacteria to acquire resistance. The resistance gene region of pEcE.ESBL.COL-01 is approximately 10 kb in size, including *dfrA14, sul2, aph(3*″*)-Ib, aph(6)-Id*, and *bla*_TEM − 1B_. The resistance region on pEcE.ESBL.COL-02 of 6.6 kbp encodes *sul2, fosA3, aph(3*″*)-Ib, aph(6)-Id, tet(A), floR*, *bla*_TEM − 1B_, and *bla*_CTX − M−55_ (Figures 5, 6). Among these, *bla*_TEM − 1B_ is a classic broad-spectrum β-lactamase gene (found in pEcE.ESBL.COL-01 and pEcE.ESBL.COL-02), while *bla*_CTX − M−55_ is an extended-spectrum β-lactamase (found in pEcE.ESBL.COL-01). (Castanheira et al., 2021). Interestingly, *bla*_CTX − M−55_ can be horizontally transferred to *E. coli* 25DN (verified by selective medium and PCR amplification, the conjugal transfer efficiency was about 0.92~2.03 × 10^−4^ CFU/donor, shown in Supplementary Figure S2 and Supplementary Table S8).

Interestingly, the pEcE.ESBL.COL-01 carried two type II toxin-antitoxin (TA) systems, *vapBC* (*vapB*: ACYG3R_24320/*vapC*: ACYG3R_24325) and *ccdAB* (*ccdA*: ACYG3R_24630/*ccdB:* ACYG3R_24625), which benefits maintaining plasmid stability (Ogura and Hiraga, 1983). The plasmid pEcE.ESBL.COL-02 carries one TA system, *pemIK* (*pemI*: ACYG3R_24790/*pemK*: ACYG3R_24795). In addition, these resistance genes are usually carried by IncFII-type plasmids, determined as the most prevalent conjugative plasmids in the Enterobacteriaceae family (Rozwandowicz et al., 2018). These observations also suggested that IncFII plasmids may potentially acquire antimicrobial resistance genes through recombination/rearrangement events, while the TA systems harbored by these plasmids could contribute significantly to the maintenance of the genetic stability (Wu et al., 2020).

### 3.5 Effects of colistin combined with LBP or NAC on ROS levels

**Figure 7:**
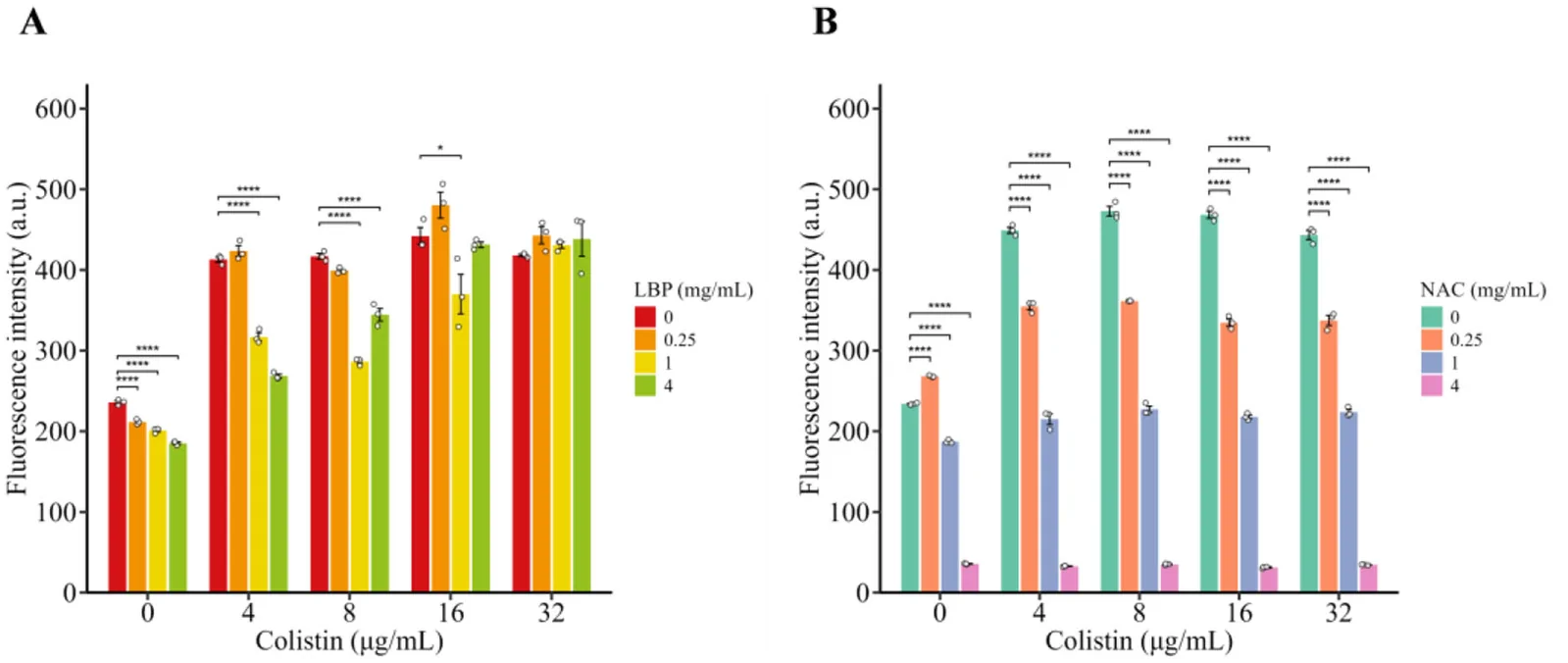
ROS levels of EcE.ESBL.COL when colistin is combined with LBP or NAC. **(A)** ROS levels of EcE.ESBL.COL treated by colistin and LBP. **(B)** ROS levels of EcE.ESBL.COL treated by colistin and NAC. ROS were detected using DCFH-DA fluorescent probes (excitation wavelength 488nm, emission wavelength 525nm). The statistical difference was assessed by using one-way analysis of variance (ANOVA) (*P < 0.05; **P < 0.01; ***P < 0.001; ****P < 0.0001).

To investigate the effect of colistin combined with LBP or NAC on the ROS levels of EcE.ESBL.COL, the DCFH-DA ROS fluorescent probe to applicable measuring the ROS levels of EcE.ESBL.COL after treatment with different concentrations of colistin combined with LBP or NAC. Interestingly, LBP could significantly reduce the ROS levels of EcE.ESBL.COL if the colistin concentration was below 1 × MIC, while LBP had no significant effect on the ROS levels of EcE.ESBL.COL if the colistin concentration was above 1 × MIC (Figure 7A). In contrast, NAC could significantly reduce the ROS levels of EcE.ESBL.COL, if NAC was combined with different concentrations of colistin (Figure 7B).

### 3.6 Comparative transcriptomics and RT-qPCR for antimicrobial determinants

The checkerboard assay and time-kill curve indicate that both LBP and NAC have no significant effect on colistin to some extent. RNA-seq technology was applicable to the identification of the effect of colistin combined with LBP and NAC on the transcription levels of EcE.ESBL.COL. The results of sample correlation analysis indicated high biological reproducibility within groups and substantial differences between different groups (Supplementary Figure S3A). Principal component analysis showed that PC1 and PC2 accounted for 90% and 7% of the total variance, respectively, and different treatment groups were clearly separated along PC1 and PC2 (Supplementary Figure S3B), further verifying the reliability of the transcriptome data. Differential expression gene analysis was performed using DESeq2, and differential expressed genes (DEGs) were screened (p_*adj*_ < 0.05, |log_2_FoldChange| >1).

A total of 1,524 DEGs were identified in the LBP + colistin group compared with the colistin group, including 712 upregulated genes and 812 downregulated genes (Supplementary Figure S3C). In the NAC + colistin group, 267 DEGs were identified, including 153 upregulated genes and 114 downregulated genes (Supplementary Figure S3D). There were 74 upregulated DEGs and 63 downregulated DEGs in both the LBP + colistin group and the NAC + colistin group (Supplementary Figure S3E). The heatmap shows the dynamic expression patterns of all DEGs across different treatment groups (Supplementary Figure S3F).

GO functional enrichment analysis and KEGG pathway enrichment analysis were performed on the upregulated genes in the LBP + colistin group. GO functional enrichment analysis indicated that the upregulated differential genes are highly related to protein synthesis (Supplementary Figure S4A). However, further KEGG pathway analysis showed that these upregulated differential genes are mainly classified into the Ribosome, Aminoacyl-tRNA biosynthesis, Flagellar assembly, Bacterial chemotaxis, Protein export, Sulfur metabolism, and Purine metabolism pathways (Supplementary Figure S4B). As a result, we assumed that LBP may defend the damage resulting from colistin by promoting the synthesis of bacteria-related proteins.

Likewise, functional enrichment analysis based on GO categories and KEGG pathways was performed on the DEGs upregulated in the NAC polymyxin group. GO-driven analysis indicated that these DEGs are related to bacterial motility, chemotaxis, signal response, and regulation functions (Supplementary Figure S4C). And KEGG analysis showed that the upregulated differential genes were significantly enriched in the Flagellar assembly, Bacterial chemotaxis, and Two-component system pathways (Supplementary Figure S4D). In addition, we assessed that NAC enhances bacterial drug resistance by affecting bacterial biofilms and two-component signal transduction systems.

Accordingly, a series of selected DEGs likely related to colistin resistance found in the transcriptome (Supplementary Table S1) were verified by RT-qPCR. Compared with the colistin-alone group, the colistin + LBP group and the colistin + NAC group both showed upregulation trends in *eptA, arnT, lptA*, and *tssB* (Supplementary Figure S5). The upregulation of *eptA* and *arnT* (involved in LPS modification, Olaitan et al., 2014), *lptA* (related to LPS transport, Klausnitzer et al., 2025), and *tssB* (associated with bacterial competitive advantages and environmental adaptability, Li et al., 2024) was found, suggesting that both LBP and NAC could enhance bacterial resistance to colistin in strain EcE.ESBL.COL.

### 3.7 Lipid A modification determination

This strain is highly resistant to polymyxin, with no *mcr* detected on the chromosome and plasmid. However, an *arn* operon was identified on the chromosome, which conferred a high-level resistance to both polymyxin B and colistin, with MIC values as high as 32 μg/mL and 16 μg/mL, respectively. This resistance level is substantially higher than the previously reported MIC values of 4–8 μg/mL colistin for *E. coli* (Yan et al., 2007). In order to investigate the discrepancy of polymyxin resistance in this strain, we analyzed the co-linearity of the *arn* operon strain EcE.ESBL.COL compared with strains CFSAN061771, KO11FL, 042, CFT073 and APEC O1 (Figure 4A and Supplementary Table S7). In contrast, except for plasmid-bearing *mcr-1*, the *arn* operon in strain CFSAN061771 was also found to likely contribute to polymyxin resistance (Hammad et al., 2022). Based on our findings, we proposed that the collaboration between *arn* operon and O-antigen variation co-operate synergistically to modify and reduce the negative charge of the outer membrane (Yan et al., 2007, Figure 4B). This alteration compromises the initial binding of polymyxin to LPS, thereby augmenting bacterial resistance (Lerouge and Vanderleyden, 2002).

**Figure 8:**
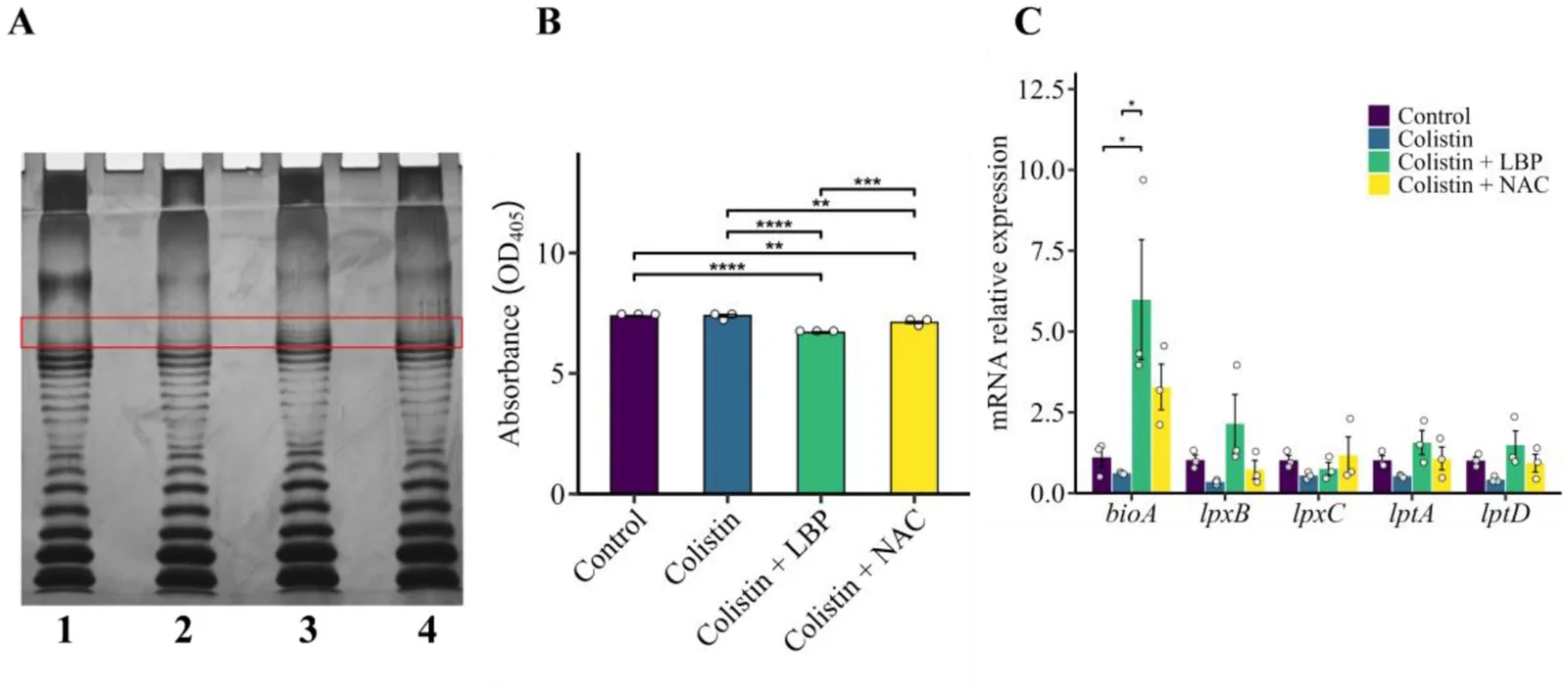
Lipopolysaccharide analysis. **(A)** SDS-PAGE analysis. Lipopolysaccharide samples were subjected to SDS-PAGE using a 5% stacking gel and a 12% resolving gel, with electrophoresis performed at 50 V for 20 min followed by 100 V for 80 min. Lane 1: control group without additional antimicrobial; Lane 2: colistin group; Lane 3: LBP + colistin group; Lane 4: NAC + colistin group. **(B)** Lipopolysaccharide content analysis. The *Limulus amebocyte* lysate (LAL) colorimetry was used, and the absorbance was measured at 405 nm. **(C)** Transcriptional changes of genes related to lipopolysaccharide synthesis. RT-qPCR analysis was performed using SYBR Green dye and a real-time PCR instrument, with the primers listed in Supplementary Tables S1, S2. The 16S rRNA gene was used as an internal reference, and the relative expression levels of the target genes were calculated using the 2^−ΔΔ*Ct*^ method. The results were presented as mean ± SE. The statistical difference was assessed by using one-way analysis of variance (ANOVA) (*P < 0.05; **P < 0.01; ***P < 0.001; ****P < 0.0001).

To investigate the dynamics of the *arn* operon in our strain, we compared the LPS profiles of the strain EcE.ESBL.COL, control strain ATCC 25922, and previously-identified LPS-deficient mutant. As a result, LPS was extracted, and transferred to SDS-PAGE analysis. Furthermore, a variety of differences in the LPS electrophoretic pattern under polymyxin treatment was found compared to that of the control, representing typical modification by L-Ara4N (no such alterations observed in the control strain). After combinatorial treatment upon these strains, the intensity of separated bands in response to the LPS architecture of strain EcE.ESBL.COL significantly enhanced, indicating an induced production of lipid A and O-antigen (Figure 8A). We also found that the content of dissociative LPS likely resulting from membrane damages was dynamic amongst groups treated by colistin alone and combined LBP/NAC (Figure 8B). This assessment was verified by using RT-qPCR for serial genes related to LPS synthesis and assembly (Figure 8C and Supplementary Table S5). Together, our findings demonstrated that the transcription of *arn* operon was activated under polymyxin stress, and further contributed to LPS modification, thereby reducing the binding capacity of LPS to polymyxin (Olaitan et al., 2014).

## 4 Discussion

In this study, we isolated a MDR *E. coli* strain EcE.ESBL.COL, of which notable phenotypic characteristic is concurrent resistance to ESBL and polymyxin. Based on comparative genomics of strain EcE.ESBL.COL and other representative *E. coli* strains, further investigation illustrated the distinct profiles for several genetic determinants related to O-antigen, virulence factors and antibiotic resistance. Together, these findings highlighted the divergent pattern of antibiotic resistance or environmental adaptability even in isolates of the same ST, resulting from the horizontal acquisition and intrinsic heterogenicity of diverse virulence and resistance determinants.

Meanwhile, pEcE.ESBL.COL-01 and pEcE.ESBL.COL-02 harbor resistance determinants, such as genes encoding β-lactamases (such as *bla*_TEM − 1B_ and *bla*_CTX − M−55_) and aminoglycoside-modifying enzymes (such as *aph(3*″*)-Ib* and *aph(6)-Id*). This study provides novel insights into the genetic mechanisms underlying the co-occurrence of ESBL and polymyxin resistance genes. Both these two plasmids belong to the IncF family, identified as one of the most predominant and highly conjugative plasmid types in *Enterobacteriaceae* and also serves as one of the most prevalent vehicles for MDR in Gram-negative bacteria (Yang et al., 2015; Rozwandowicz et al., 2018). In general, IncF plasmids exhibit strong genetic plasticity, enabling them to integrate other replicons and functional genes to constitute hybrid plasmids. Furthermore, serial toxin-antitoxin systems and antibiotic resistance genes was found, likely enhancing plasmid stability and host adaptability (Mathers et al., 2015).

Comparative analysis of the virulence factors and resistance genes located on both the chromosome and plasmids revealed their genomic contexts with distinct patterns. This finding suggests that virulence factors and resistance genes may suffer relatively independent evolution, indicating that the acquisition of these modular elements is not only related to sequence type and chromosomal determinants, but also is more likely attributable to horizontal transfer mediated by mobile genetic elements (Tokuda and Shintani, 2024). In other words, horizontal gene transfer could accelerate the spread and convergence of antibiotic resistance genes (Zhou et al., 2021).

Generally, the O-antigen functions not only as a virulence factor and a major component of LPS, but also likely serves as a recognition target for antimicrobial peptides. According to literature reports, *arn* operon mediated resistance to polymyxins is generally considered to be inducible (previously reported colistin MIC for *E. coli* was 4–8 μg/mL; Yan et al., 2007; Zhang et al., 2019; Lu et al., 2022). Therefore, the diversity of polymyxin resistance prompted us to reveal that the O-antigen pertaining to LPS differed compared to another ST1485 strain. It is feasible that Arn-driven alterations and the O-antigen reconfiguration can affect the conformation of LPS, thereby likely influencing the electrostatic interaction and hinderance barriers between antimicrobial peptides and LPS, which contributes to the development of bacterial resistance to these peptides (Lerouge and Vanderleyden, 2002; Chin et al., 2021; Lu et al., 2025). Therefore, we speculated that the modification of the O antigen could contribute synergistically to the enhanced polymyxin resistance resulting from *arn* operon.

Nevertheless, strain EcE.ESBL.COL exhibited high MIC of 16 μg/mL for colistin. Genomic analysis and LPS architecture typing confirmed that polymyxin resistance in EcE.ESBL.COL is mediated by chromosomal *arn*-dependent L-Ara4N modification and O-antigen reconfiguration in LPS. It is noteworthy that polysaccharide LBP and antioxidant NAC could augment or has no effect the polymyxin resistance of strain EcE.ESBL.COL (no significant antimicrobial coordination if combined with colistin), although a series of changes in gene transcription and LPS architectures were observed (Supplementary Figure S6). This finding indicated that the ubiquitous diversity in effects and targets of natural products or artificial intermediates in reversing polymyxin resistance (Chai et al., 2020; Cai et al., 2023; Zhong et al., 2023; Cheng et al., 2024; Zhao et al., 2026). Collectively, the LPS assembly/dissociation, modification determinants, oxidative stress and membrane integrity should be sufficiently measured *in vitro*, benefiting the directed control of polymyxin resistance by using additional adjuvants (Diao et al., 2025; Zhao et al., 2026).

In conclusion, a polymyxin-resistant *Escherichia coli* strain, designated EcE.ESBL.COL, was isolated from a urine sample. Whole-genome sequencing analysis revealed that the strain carries one chromosome and three plasmids, belonging to ST1485 and serotype O25:H42. Further, comparative genomics identified multiple resistance genes associated with ESBL production, specially a MDR integron-like region on the chromosome, *bla*_TEM − 1B_ on pEcE.ESBL.COL-01, and *bla*_TEM − 1B_/*bla*_CTX − M−55_ on pEcE.ESBL.COL-02. Concerning that the polymyxin resistance-related *arn* operon and serial LPS related dynamics by concomitant treatment, we assumed that the observed high-level polymyxin resistance is mainly attributed to the LPS architectural modification mediated by this *arn* operon together with the distinctly-related O-antigen variation, by corresponding genomic analysis and SDS-PAGE. Collectively, our finding indicated the diversity of polymyxin resistance related to LPS structural modification, and provided a comparative insight into the co-occurrence of ESBL and Arn determinants if encountering the “trade-off” by additional adjuvants.

## Data Availability

The original contributions presented in the study are included in the article/Supplementary material, further inquiries can be directed to the corresponding authors.
