# Supplementary material for: Spread potential and architectural variant of a multiple-drug resistant Escherichia coli co-producing lipopolysaccharide-modifying determinants and extended-spectrum β-lactamases
Source: Front Microbiol. 2026 Sep 8;17:1924788. doi: 10.3389/fmicb.2026.1924788 (PMC13593837; doi:10.3389/fmicb.2026.1924788)
Supplement: Supplementary file 1 [file Data_Sheet_1.pdf]

Figure S1. Growth curves of strain EcE.ESBL.COL under different stresses

Figure S2. PCR-amplified DNA fragments of *bla*$_{CTX\text{-}M\text{-}55}$ and *bla*$_{TEM\text{-}1B}$

Figure S3. Transcriptomic changes induced by colistin combined with LBP or NAC

Figure S4. Functional analysis of differentially expressed genes

Figure S5. RT-qPCR verification of genes related to lipopolysaccharide synthesis and two-component signal transduction system

Figure S6. Schematic of colistin combined with LBP and NAC

Table S1. Differentially expressed genes selected from the transcriptome

Table S2. Primers of gene transcription and transconjugant verification

Table S3. General genomic information of strains EcE.ESBL.COL and CFSAN061771

Table S4. Serotyping for EcE.ESBL.COL and five representative strains

Table S5. Comparison of chromosomal virulence factors in strains EcE.ESBL.COL, CFSAN061771, KO11FL, 042, CFT073 and APEC O1

Table S6. Comparison of virulence factors in pEcE.ESBL.COL-01 and pCFSAN061771_01

Table S7. Comparative analysis of polymyxin resistance-associated lipopolysaccharide (LPS) modification enzymes across five strains

Table S8. Viable counts of donor strains (ECE.ESBL.COL), transconjugants (25DN-Amp), and the calculated conjugation transfer frequencies

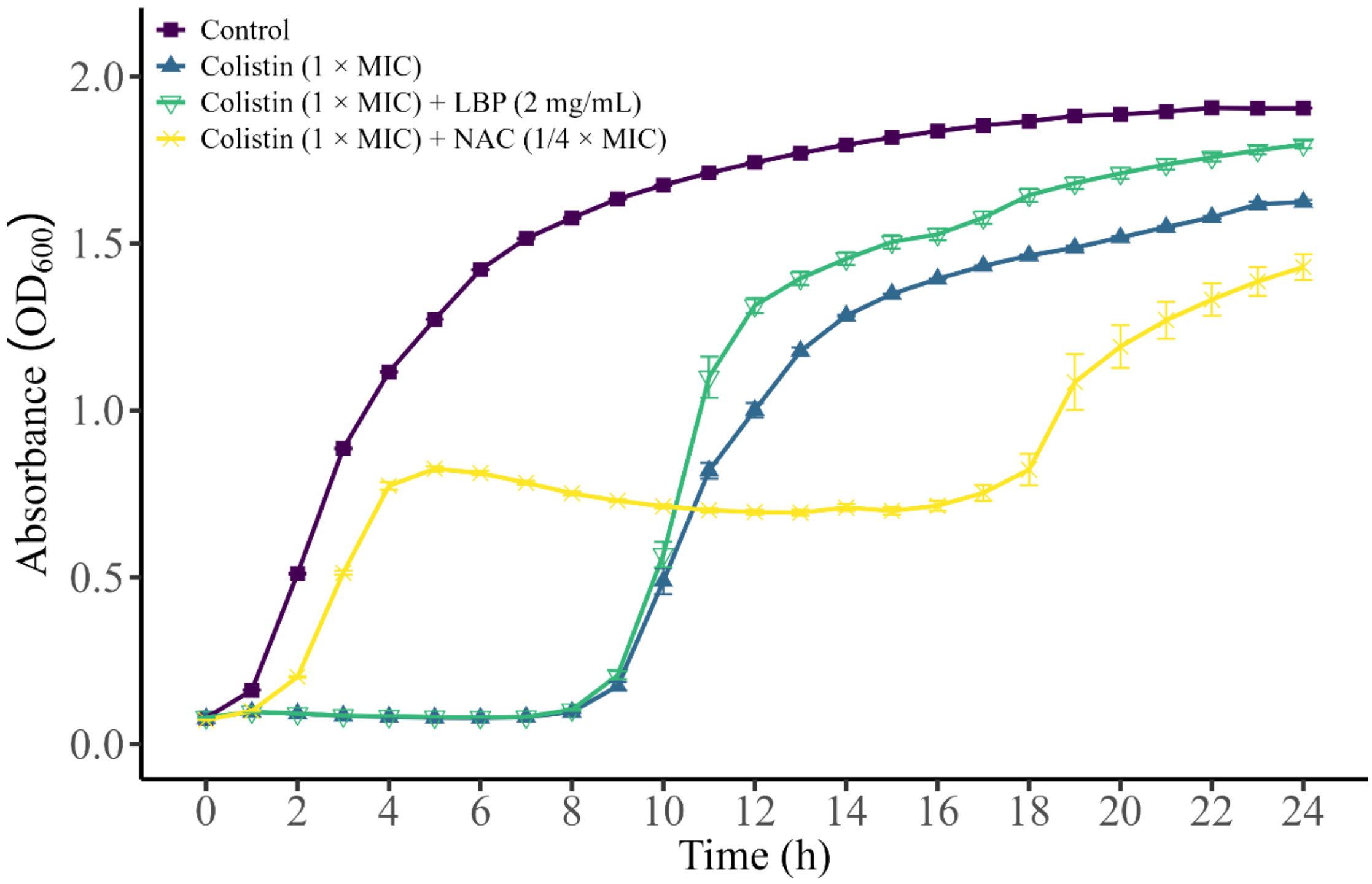

Figure S1. Growth curves of strain EcE.ESBL.COL under different stresses. EcE.ESBL.COL was cultured under different treatment conditions at 37 °C with shaking at 180 rpm for 24 h and the OD_{600} was measured every hour. The corresponding results were presented as mean ± SE.

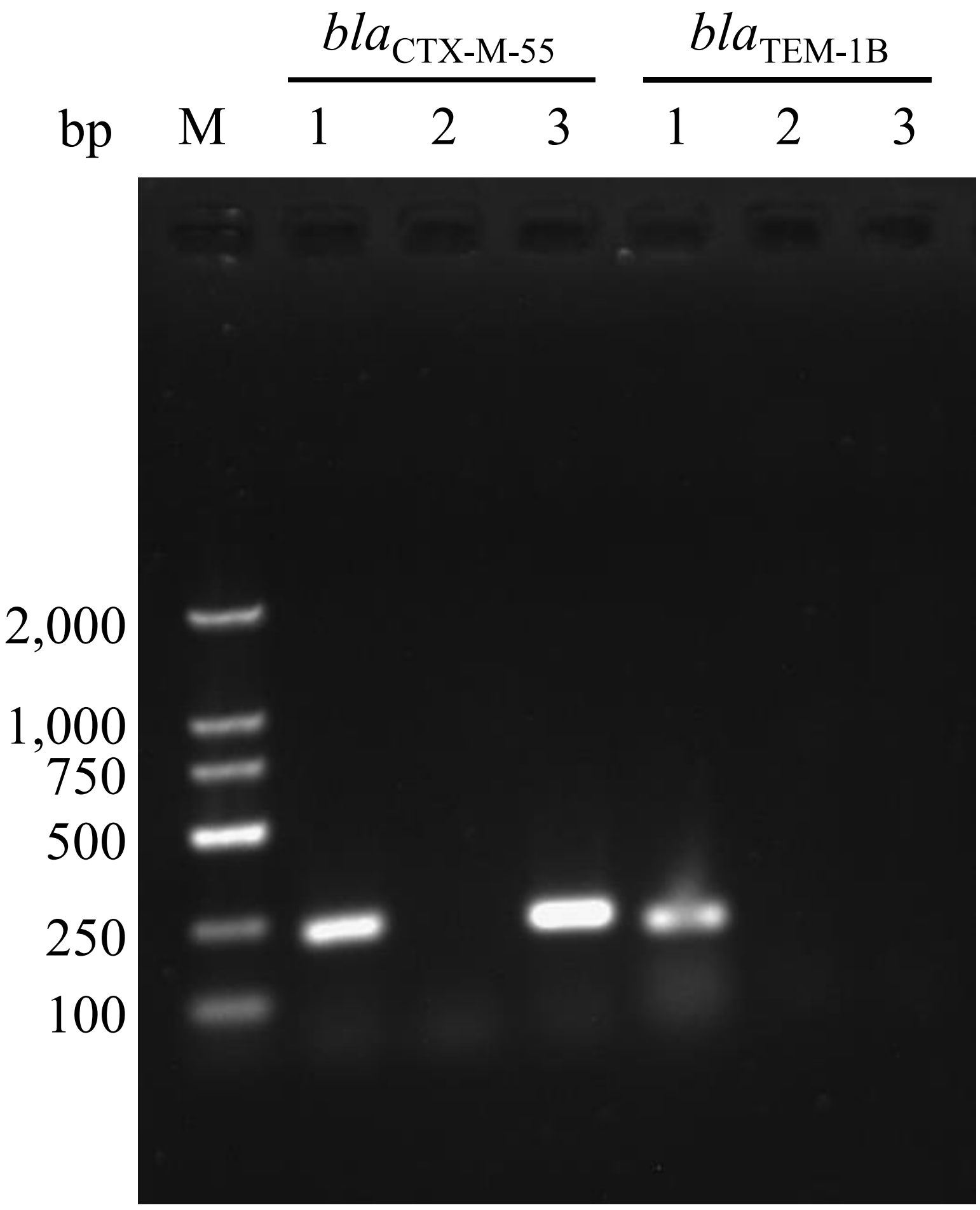

Figure S2. PCR-amplified DNA fragments of *bla*$_{CTX-M-55}$ and *bla*$_{TEM-1B}$. Note: lane 1, total DNA from strain ECE.ESBL.COL; lane 2, strain 25DN; and lane 3, transconjugant 25DN-Amp.

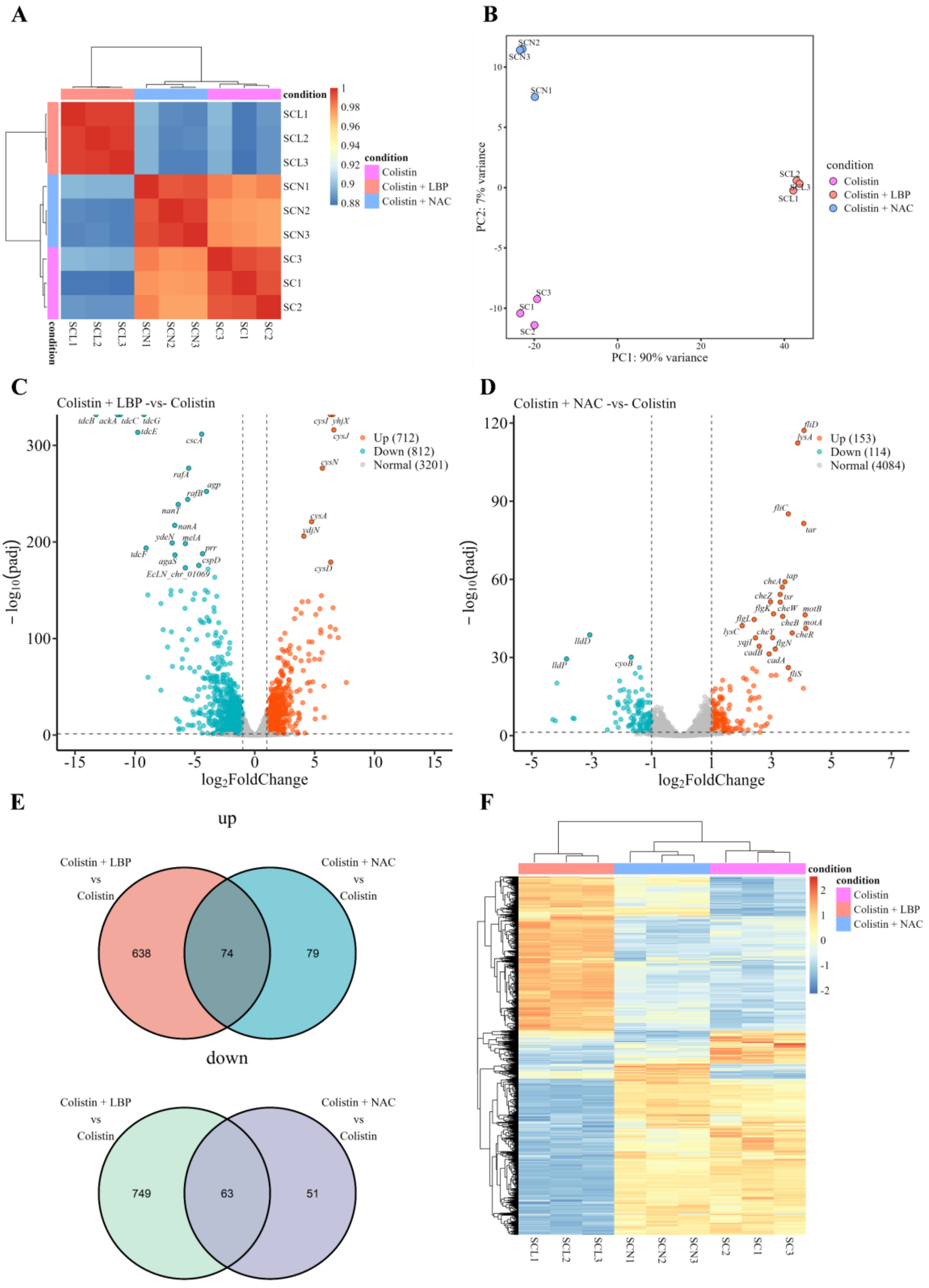

Figure S3. Transcriptomic changes induced by colistin combined with LBP or NAC. (**A**) Correlation heatmap. (**B**) Principal component analysis (PCA). (**C**) Volcano plot for colistin + LBP vs. colistin. (**D**) Volcano plot for colistin + NAC vs. colistin. Red: upregulated genes; blue: downregulated genes; gray: not significant ($|log_2FoldChange| < 1$ or $p_{adj} > 0.05$). (**E**) Venn diagram showing the number of up- and down-regulated DEGs. (**F**) Heatmap showing the expression patterns of all DEGs. Red and blue denote gene upregulation and downregulation, respectively.

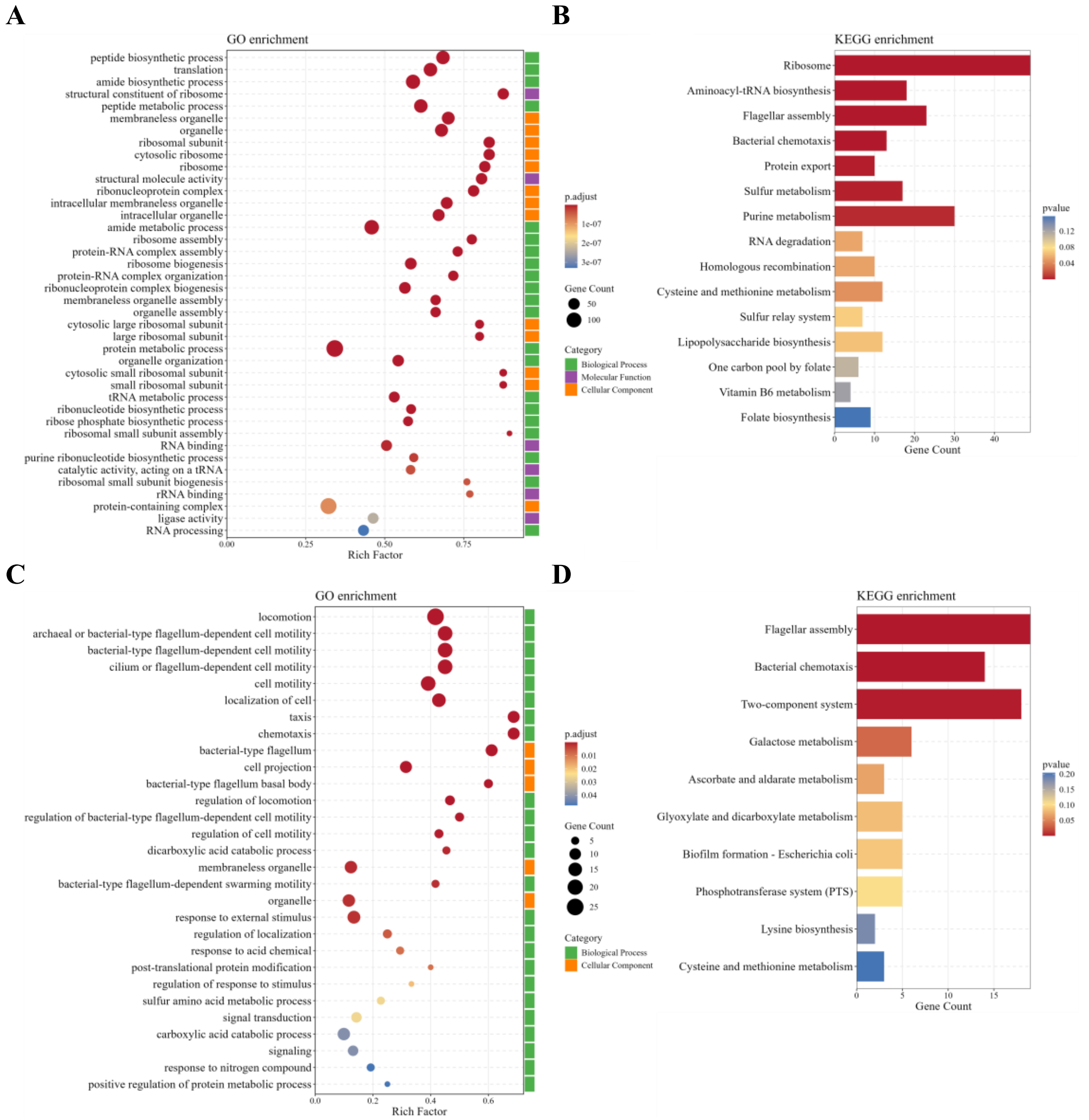

Figure S4. Functional analysis of differentially expressed genes. (**A**) GO and (**B**) KEGG categories of up-regulated genes in LBP + colistin group. (**C**) GO and (**D**) KEGG categories of up-regulated genes in NAC + colistin group.

A

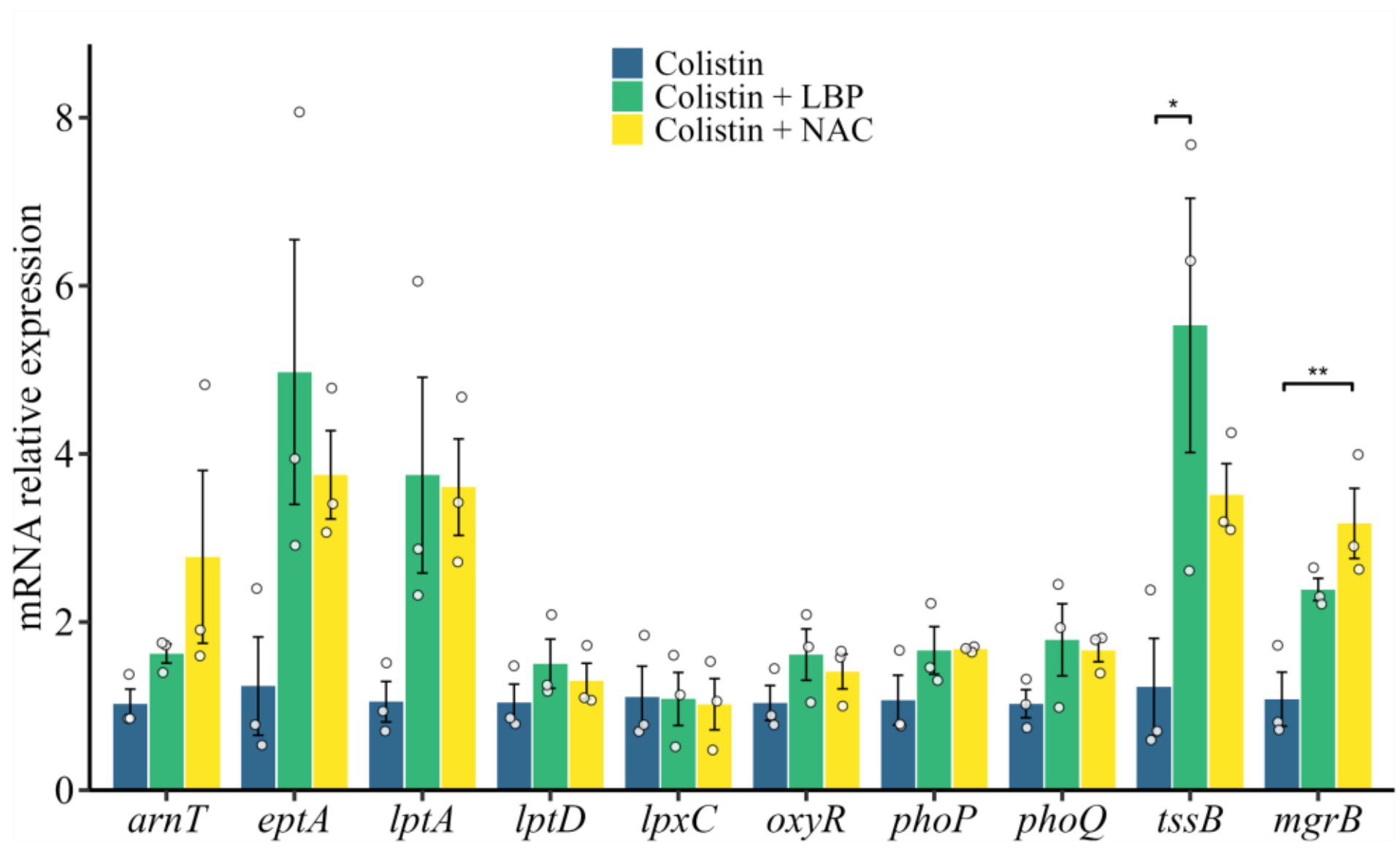

B

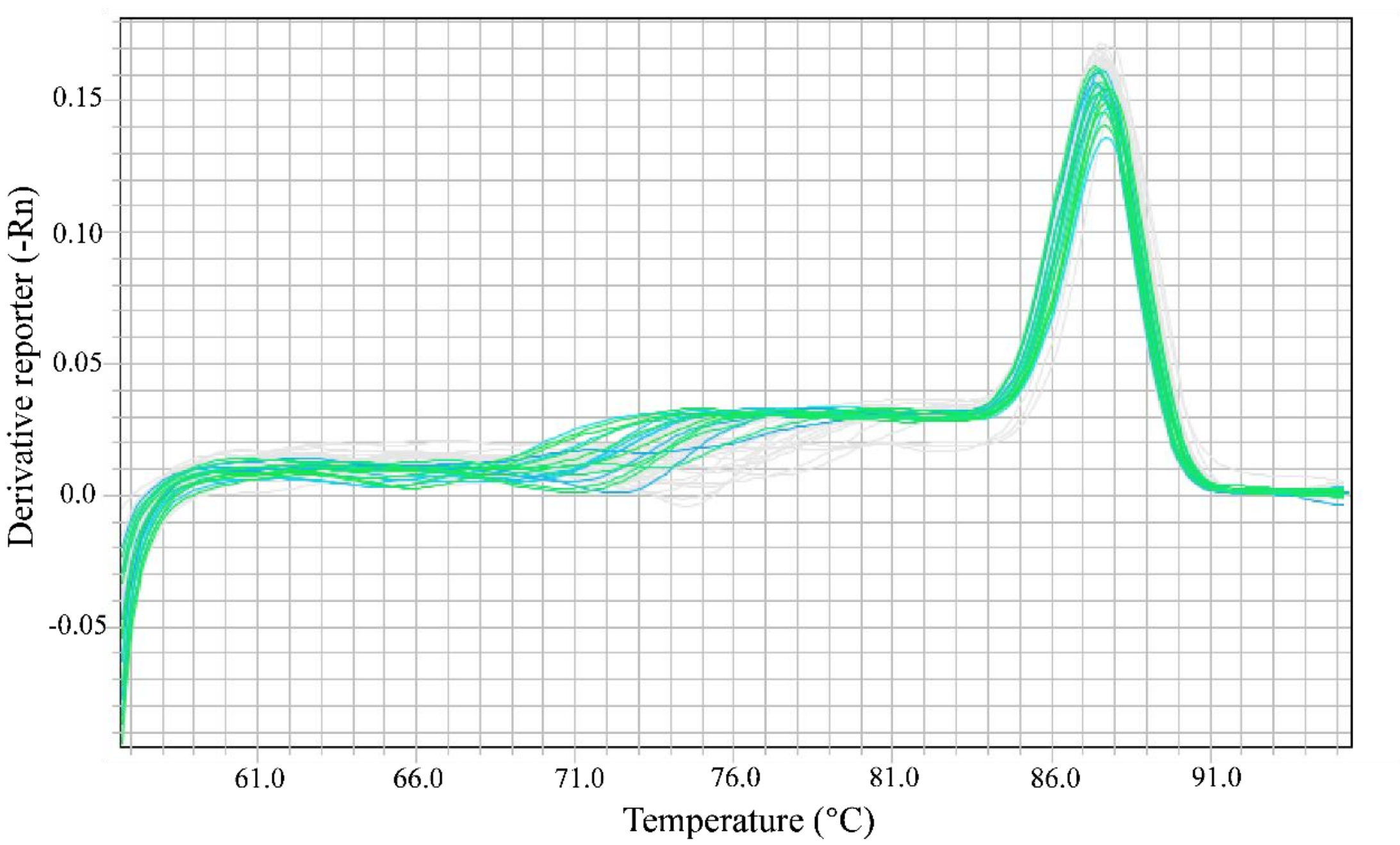

Figure S5. RT-qPCR verification of genes related to lipopolysaccharide synthesis and two-component signal transduction system. (**A**) Gene transcriptional difference. RT-qPCR analysis was performed using SYBR Green dye and a real-time PCR instrument. The 16S rRNA gene was used as an internal reference, and the relative expression levels of the target genes were calculated using the $2^{-\Delta\Delta Ct}$ method. The results were presented as mean ± SE. The statistical significance of differences among groups was assessed using one-way analysis of variance (ANOVA) (*, P < 0.05; **, P < 0.01; ***, P < 0.001; ****, P < 0.0001). (**B**) Melting curves of the 16S rRNA (internal reference) gene by quantitative PCR with varying concentrations of cDNA templates.

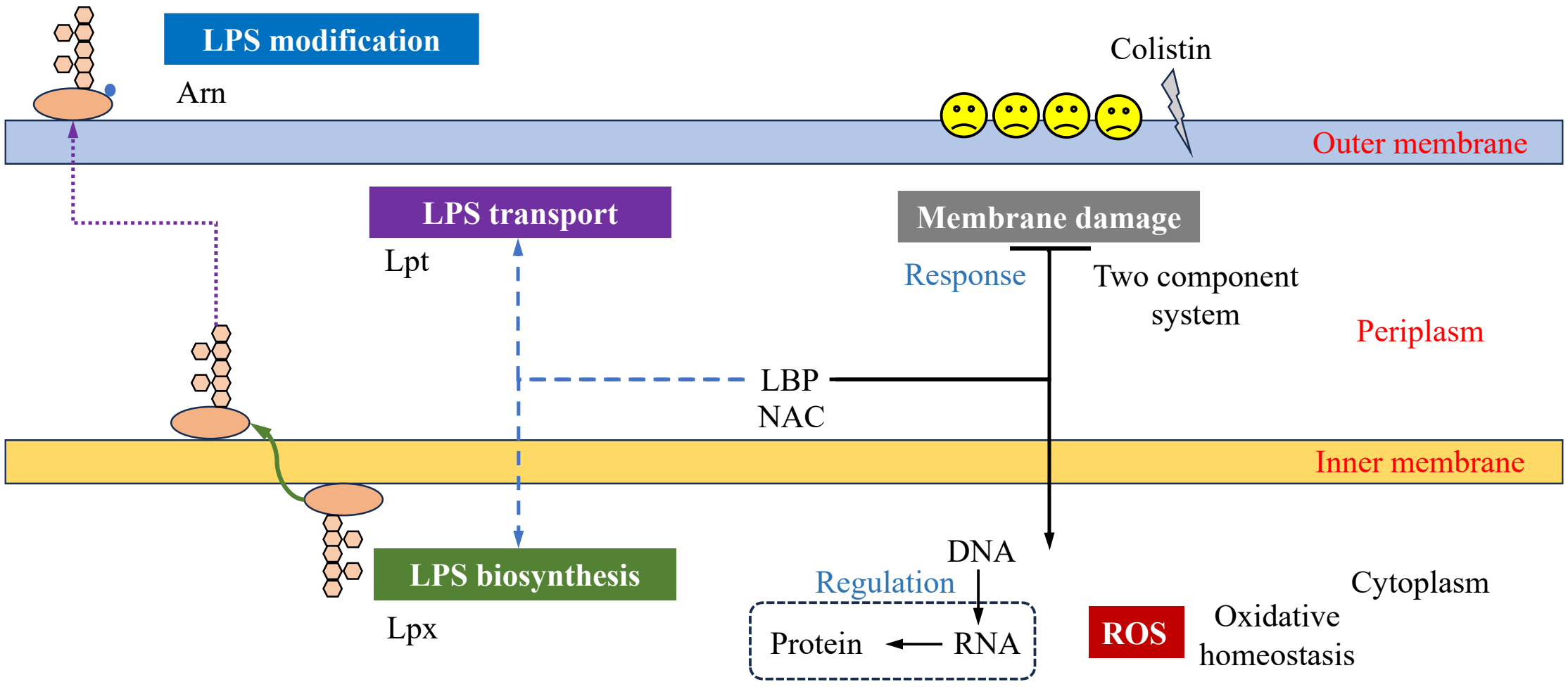

Figure S6. Schematics of intracellular homeostasis accommodation if encountering colistin combined with LBP and NAC. Note: LPS, lipopolysaccharide; ROS, reactive oxygen species; LBP; *Lycium barbarum* polysaccharides; NAC, N-acetylcysteine.

Table S1. Differentially expressed genes selected from the transcriptome

Locus tag	Description	Gene	Colistin + LBP / Colistin			Colistin + NAC / Colistin		
			log2FC	p-value	Regulation	log2FC	p-value	Regulation
ACYG3R_08785	Undecaprenyl phosphate-alpha-4-amino-4-deoxy-L-arabinose arabinosyl transferase	*arnT*	-0.0654	0.7043	down	-0.3729	0.031677	down
ACYG3R_22390	Phosphoethanolamine transferase EptA	*eptA*	1.4837	7.71E-36	up	0.4214	4.44E-04	up
ACYG3R_03490	Lipopolysaccharide export system protein LptA	*lptA*	0.3094	5.06E-04	up	0.2002	0.024463	up
ACYG3R_20320	LPS-assembly protein LptD	*lptD*	0.3249	3.22E-04	up	0.4518	5.53E-07	up
ACYG3R_20100	UDP-3-O-acyl-N-acetylglucosamine deacetylase	*lpxC*	-0.2149	0.008465	down	0.1353	0.096806	up
ACYG3R_02875	Hydrogen peroxide-inducible genes activator	*oxyR*	-0.7343	2.65E-10	down	-0.4673	5.57E-05	down
ACYG3R_14610	Transcriptional regulatory protein PhoP	*phoP*	-0.5699	3.63E-07	down	-0.2368	0.026487	down
ACYG3R_14615	Sensor protein PhoQ	*phoQ*	0.1653	0.101662	up	0.3988	6.76E-05	up
ACYG3R_19395	Hypothetical protein	*tssB*	1.1944	1.28E-08	up	-0.4994	0.033216	down

Note: FC, fold change; LBP; *Lycium barbarum* polysaccharides; NAC, N-acetylcysteine.

Table S2. Primers for gene transcription and transconjugant verification

Gene	Forward (5'-3')	Reverse (5'-3')	Product length (bp)
RT-qPCR			
16S	TCTTTGTATGCGCCATTGTA	ACCCTTATCCTTTGTTGCC	144
arnT	ACTGATAGCGTCGGTGTTGC	ACCGCTGACAAATCTCCCTT	132
eptA	TCACCGTGGTCAGAAAGAT	CCTGTACCCAAGAGCAACT	142
lptA	GATCAGCCGATCCACATT	CGGGTAACGACCACTTTG	131
lptD	CCATATCGCGTTCAAAGAC	TGGCAACCCATTATCAGC	132
lpxC	CGGATTTCAACCGTAGAGC	GGCGTCAAGCAGCAGGTA	138
oxyR	CCAATAGTGCGGCGAGGTT	ACATGGTGGCGGCAGGTAG	121
phoP	CACTTTGCCATTATTGCGTAT	GGCTTCACAGGTCATTTCG	139
phoQ	GCAATTCGCGGTTATGTTC	TGGTCTGGAGCTGGTTTATCT	147
mgrB	GGATAAACTGGTTAATGGCA	ATGGGTCGTTCTGGTTGT	122
tssB	TGGCCGATTTCTTTGAGC	GAACTGCGTGAAGCACTGACT	125
lpxB	GCTCGCATTTCTGCCTTTC	GCACATCACGGGCACCAT	125
bioA	GGCGGAAAGGGTCATTGTG	TTGCTGATTGCCGACGAGA	135
PCR for transconjugant verification			
$bla_{\text{CTX-M-55}}$	CAGATTCGGTTCGCTTTCAC	CCGTCACGCTGTTGTTAGGA	233
$bla_{\text{TEM-1B}}$	ATACCGCACCACATAGCAG	GTCGCCCTTATTCCCTTTT	214

Table S3. General genomic information of strains EcE.ESBL.COL and CFSAN061771

EcE.ESBL.COL	MLST/Inc	Size (bp)	G+C (%)	ARG	CFSAN061771	MLST/Inc	Size (bp)	G+C (%)	ARG
chromosome	ST1485	5,039,821	50.46	*folP* (ACYG3R_05245)<br>*aph(3'')-Ib* (ACYG3R_05250)<br>*aph(6)-Id* (ACYG3R_05255)<br>*tet(A)* (ACYG3R_05265)<br>*floR* (ACYG3R_05280)	chromosome	1485	4,908,204	50.58	-
pEcE.ESBL.COL-01	IncFIA<br>IncFIB (AP001918)<br>IncFIC(FII)	167,587	50.41	*dfrA14* (ACYG3R_24205)<br>*sul2* (ACYG3R_24235)<br>*aph(3'')-Ib* (ACYG3R_24240<br>*aph(6)-Id* (ACYG3R_24245)	pCFSAN061771_01	IncFIA<br>IncFIB<br>IncFIC (FII)	167,754	50.37	bla_{TEM-1B} (CA902_023455)<br>*aph(6)-Id* (CA902_023465)<br>*aph(3'')-Ib* (CA902_023470)<br>*sul2* (CA902_023475)

				bla$_{\text{TEM-1B}}$<br>(ACYG3R_24250)					*dfrA14*<br>(CA902_023515)
pEcE.ESBL.COL-02	FII	78,689	52.99	*folP*<br>(ACYG3R_24815)<br>*aph(3'')-Ib*<br>(ACYG3R_24820)<br>*aph(6)-Id*<br>(ACYG3R_24825)<br>*tet(A)*<br>(ACYG3R_24840)<br>*floR*<br>(ACYG3R_24855)<br>*fosA3*<br>(ACYG3R_24890)<br>*bla*$_{\text{TEM-1B}}$<br>(ACYG3R_24900)<br>*bla*$_{\text{CTX-M-55}}$<br>(ACYG3R_24910)	pCFSAN06177<br>1_02	IncHI2<br>IncHI2A	215,531	45.27	*sul3*<br>(CA902_024570)<br>*aadA*<br>(CA902_024590)<br>*cmlA1*<br>(CA902_024595)<br>*aadA2b*<br>(CA902_024600)<br>*mcr-1.1*<br>(CA902_024940)
pEcE.ESBL.COL-03	-	1998	40.89	-					

Note: MLST, multi-locus sequence types; Inc, Incompatible groups.

Table S4. Serotyping for EcE.ESBL.COL and five representative strains

Strain (Genome accession No.)	MLST	Gene number	Size (bp)	Flagellar antigen	Serotype	Identity (%)	O antigen genes	Serotype	Identity (%)	Plasmid (size (bp)/ORF)	Plasmid accession No.
EcE.ESBL.COL	1485	4,674	5,039,821	*fliC*	H42	98.67	*wzx*	O25	99.92	167,587/179	JBTWAA010
(JBTWAA010000001)							*wzy*		99.91	78,689/96	000002-4
										1998/4	
E. coli strain CFSAN061771	1485	4,657	4,908,204	*fliC*	H42	98.67	*wzy*	O83	99.92	167,754/139	CP042897
(CP042896)										215,531/139	CP042898
E. coli strain KO11FL	1079	4,813	5,021,812	*fliC*	H49	100	*wzy*	O6	100	5,360/12	CP002971
(CP002970)											
E. coli strain 042	410	4,569	4,692,707	*fliC*	H21	99.53	-	-	-	51,479/69	CP042935
(CP042934)										45,674/72	CP042936
E. coli strain CFT073	73	5,590	5,231,428	*fliC*	H1	100	*wzx*	O6	100	-	-
(NC_004431)											
E. coli strain APEC O1	95	4,560	5,082,025	*fliC*	H7	100	*wzx*	O1	99.8	241,387/224	DQ517526
(CP000468)							*wzy*		99.84	174,241/199	DQ381420

Note: MLST, multi-locus sequence types; ORF, open reading frame.

Table S5. Comparison of chromosomal virulence factors in strains EcE.ESBL.COL, CFSAN061771, KO11FL, 042, CFT073 and APEC O1

VF class	Virulence factors	Related genes	Strain EcE.ESBL.COL	Strain CFSAN061771	Strain KO11FL	Strain 042	Strain CFT073	Strain APEC O1
			JBTWAA010000001	CP042896	CP002970	CP042934	NC_004431	CP000468
Adherence	AAF/II fimbriae	*aafA*	-	-	-	-	-	-
		aafB	-	-	-	-	-	-
		aafC	-	-	-	-	-	-
		aafD	-	-	-	-	-	-
	AAF/III fimbriae	*agg3A*	-	-	-	-	-	-
		agg3B	-	-	-	-	-	-
		agg3C	-	-	-	-	-	-
		agg3D	-	-	-	-	-	-
	A fimbrial adhesin AFA-I	*afaA*	-	-	-	-	-	-
		afaB	-	-	-	-	-	-
		afaC	-	-	-	-	-	-
		afaD	-	-	-	-	-	-
		afaE	-	-	-	-	-	-
		draP	-	-	-	-	-	-
	CFA/I fimbriae	*cfaA*	ACYG3R_04010	CA902_003275	KO11_07220	FVF73_15850	-	-
		cfaB	ACYG3R_04015	CA902_003280	KO11_07225	FVF73_15845	-	-
		cfaC	ACYG3R_04020	CA902_003285	KO11_07230	FVF73_15840	-	-
		cfaD/cfaE	ACYG3R_04025	CA902_003290	KO11_07235	FVF73_15835	-	-
	Curli fibers	*cgsD*	-	-	-	-	C_RS06115; c1302	APECO1_125
		cgsE	-	-	-	-	C_RS06110; c1301	APECO1_124

		cgsF	-	-	-	-	C_RS06105; c1300	APECO1_123
		cgsG	-	-	-	-	C_RS06100; c1299	APECO1_122
		csgA	-	-	-	-	C_RS06130; c1306	APECO1_127
		csgB	-	-	-	-	C_RS06125; c1305	APECO1_126
		csgC	-	-	-	-	C_RS06135; c1307	-
	Dispersin	*aap*	-	-	-	-	-	-
	E. coli common pilus (ECP)	*ecpA*	ACYG3R_19060	CA902_017905	KO11_22130	FVF73_01515	C_RS01860; c0404	APECO1_1704
		ecpB	ACYG3R_19065	CA902_017910	KO11_22135	FVF73_01510	C_RS01855; c0403	APECO1_1705
		ecpC	ACYG3R_19070	CA902_017915	KO11_22140	FVF73_01505	C_RS01850; c0402	APECO1_1706
		ecpD	ACYG3R_19075	CA902_017920	KO11_22145	FVF73_01500	C_RS01845; c0401	APECO1_1707
		ecpE	ACYG3R_19080	CA902_017925	KO11_22150	FVF73_01495	C_RS01840; c0400	APECO1_1708
		ecpR	ACYG3R_19055	CA902_017900	KO11_22125	FVF73_01520	C_RS01865; c0405	APECO1_1703
	E. coli laminin-binding fimbriae (ELF)	*elfA*	ACYG3R_15515	CA902_014350	KO11_18035	FVF73_04970	-	-
		elfC	ACYG3R_15505	CA902_014340	KO11_18025	FVF73_04980	-	-
		elfD	ACYG3R_15510	CA902_014345	KO11_18030	FVF73_04975	-	-
		elfG	ACYG3R_15500	CA902_014335	KO11_18020	FVF73_04985	-	-
	EaeH	*eaeH*	ACYG3R_19000	CA902_017835	KO11_22095	-	C_RS01915; c0415	APECO1_1694
	EtpA	*etpA*	-	-	-	-	-	-
	F1C fimbriae	*focA*	-	-	-	-	C_RS05815; c1239	-
		focC	-	-	-	-	C_RS05825; C_RS09085; c1241; c1935	APECO1_633
		focD	-	-	-	-	C_RS05830; c1242	-
		focF	-	-	-	-	C_RS05835; c1243	-

		focG	-	-	-	-	C_RS05840; c1244	-
		focH	-	-	-	-	C_RS05845; c1245	-
		focI	-	-	-	-	c1240	-
	Hemorrhagic *E. coli* pilus (HCP)	*hcpA*	ACYG3R_20045	CA902_018820	KO11_00515	FVF73_00740	C_RS00595; c0127	APECO1_1881
		hcpB	ACYG3R_20050	CA902_018825	KO11_00510	FVF73_00735	C_RS00590; c0126	APECO1_1882
		hcpC	ACYG3R_20055	CA902_018830	KO11_00505	FVF73_00730	C_RS00585; c0125	APECO1_1883
	Intimin	*eae*	-	-	-	-	-	-
	K88 fimbriae	*faeC*	-	-	-	-	-	-
		faeD	-	-	-	-	-	-
		faeE	-	-	-	-	-	-
		faeF	-	-	-	-	-	-
		faeG	-	-	-	-	-	-
		faeH	-	-	-	-	-	-
		faeI	-	-	-	-	-	-
		faeJ	-	-	-	-	C_RS17040; C_RS24520; c3592; c5188	-
	P fimbriae	*papA*	-	-	-	-	C_RS17045; C_RS24525	APECO1_3519
		papB	ACYG3R_05300	-	-	-	C_RS17030; C_RS18020; C_RS24510; c3590; c3792; c5186	APECO1_3518

		papC	-	-	-	-	C_RS17025; C_RS24505; c3589; c5185	APECO1_3369; APECO1_3521; APECO1_4228
		papD	-	-	-	-	C_RS17010; C_RS24490; c3585; c5181	APECO1_3522
		papE	-	-	-	-	C_RS17005; C_RS24485; c3584; c5180	APECO1_3525
		papF	-	-	-	-	C_RS17000; C_RS24480; c3583; c5179	APECO1_3526
		papG	-	-	-	-	C_RS17035; C_RS24515; c3591; c5187	APECO1_3527
		papH	-	-	-	-	C_RS17055; C_RS24530; c3593; c5189	APECO1_3520
		papI	ACYG3R_05295	-	-	-	C_RS17020; C_RS24500; c3588; c5184	APECO1_3517
		papJ	-	-	-	-	C_RS17015; C_RS24495; c3586; c5182	APECO1_3523

		papK	-	-	-	-	C_RS05855; C_RS16995; c1247; c3582	APECO1_3524
		papX	-	CA902_004215	-	-	-	APECO1_2260
	Porcine attaching-effacing associated protein	*paa*	-	-	-	-	-	-
	S fimbriae	*sfaA*	-	-	-	-	C_RS05810; c1238	-
		sfaB	-	-	-	-	C_RS05805; c1237	-
		sfaC	-	-	-	-	C_RS05820	-
		sfaD	-	-	-	-	C_RS17040; C_RS24520; c3592; c5188	-
		sfaE	-	-	-	-	-	-
		sfaF	-	-	-	-	-	-
		sfaG	-	-	-	-	-	-
		sfaH	-	-	-	-	-	-
		sfaS	-	-	-	-	-	-
	ToxB	*toxB*	-	-	-	-	-	-
	Type I fimbriae	*fimA*	ACYG3R_21070	CA902_019835	KO11_23195	FVF73_22460	C_RS09090; C_RS25460; c1936; c5393	APECO1_2116; APECO1_634
		fimB	ACYG3R_21080	CA902_019845	KO11_23185	FVF73_22450	C_RS01990; C_RS13740;	APECO1_1681; APECO1_2118

							C_RS13745; C_RS25445; c0430; c2897; c2898; c5391	
		fimC	ACYG3R_21060	CA902_019825	KO11_23205	FVF73_22470	C_RS25470; c5395	APECO1_2114
		fimD	ACYG3R_12505; ACYG3R_21055	CA902_011335; CA902_019820	KO11_15650; KO11_23210	FVF73_07710; FVF73_22475	C_RS09080; C_RS19935; C_RS25475; c1934; c4212; c5396	APECO1_1846; APECO1_2113; APECO1_3032; APECO1_4437; APECO1_632
		fimE	ACYG3R_21075	CA902_019840	KO11_23190	FVF73_22455	C_RS25450; c5392	APECO1_2117
		fimF	ACYG3R_12510; ACYG3R_21050	CA902_011340; CA902_019815	KO11_15655; KO11_23215	FVF73_07705; FVF73_22480	C_RS09075; C_RS25480; c1933; c5397	APECO1_2112; APECO1_631
		fimG	ACYG3R_12515; ACYG3R_21045	CA902_011345; CA902_019810	KO11_15660; KO11_23220	FVF73_07700; FVF73_22485	C_RS09070; C_RS25485; c1932; c5399	APECO1_2111; APECO1_630
		fimH	ACYG3R_21040	CA902_019805	KO11_23225	FVF73_22490	C_RS25490; c5400	APECO1_2110
		fimI	ACYG3R_21065	CA902_019830	KO11_23200	FVF73_22465	C_RS25465; c5394	APECO1_2115
	Hsp60 (*Legionella*)	*htpB*	-	-	-	-	C_RS24695; c5227	-
	Lateral flagella (*Aeromonas*)	*lfhA*	-	-	-	-	c0377	-
	Polar flagella (*Aeromonas*)	*flmH*	-	-	-	-	C_RS06395	-
		nueA	-	-	-	-	C_RS04985; c1059	-

	Streptococcal plasmin receptor/GAPDH (*Streptococcus*)	*plr/gapA*	-	-	-	-	C_RS08650; C_RS10330; c1843; c2184	-
	Type IV pili (*Yersinia*)	*pilW*	-	-	-	-	C_RS00760; C_RS13220; C_RS25600; c0162; c2786; c5420	-
	LPS O-antigen (*P. aeruginosa*) (*Pseudomonas*)	-	ACYG3R_09925	-	-	-	-	-
	Lateral flagella (*Aeromonas*)	*flgC*	-	-	KO11_22360	-	-	-
Autotransporter	AIDA-I	*aida*	-	-	-	-	-	-
	AIDA-I type	*tibA*	-	-	-	-	-	-
	AatA	*aatA*	ACYG3R_21440	-	-	-	-	-
	Antigen 43	*agn43*	-	-	-	-	C_RS05985; C_RS17360; c1273; c3655	-
	Cah	*cah*	-	-	-	-	-	-
	Contact-dependent inhibition CDI system	*cdiA*	-	-	-	-	C_RS01580; c0345	-
		cdiB	-	-	-	-	-	-
	EhaA	*ehaA*	-	-	KO11_22025	-	-	-
	EhaB	*ehaB*	ACYG3R_18665	CA902_017505	KO11_21745	-	C_RS02215; c0478	APECO1_1634

	Enteroaggregative immunoglobulin repeat protein	*air/eaeX*	ACYG3R_00240	CA902_000235	-	-	-	-
	EspC	*espC*	-	-	-	-	-	-
	EspI	*espI*	-	-	-	-	-	-
	EspP	*espP*	-	-	-	-	-	-
	Pet	*pet*	-	-	-	-	-	-
	Pic	*pic*	-	-	-	-	C_RS01620; c0350	-
	Sat	*sat*	-	-	-	-	C_RS17200; c3619	-
	Temperature-sensitive hemagglutinin	*tsh*	-	-	-	-	-	-
	UpaG adhesin	*upaG/ehaG*	ACYG3R_00610	CA902_000665	KO11_04675	FVF73_18595	C_RS20895; c4424	APECO1_2853
	UpaH	*upaH*	-	-	-	-	-	APECO1_557
	Vacuolating autotransporter gene	*vat*	-	-	-	-	C_RS01810; c0393	APECO1_1712
Invasion	Invasion of brain endothelial cells (Ibes)	*ibeA*	-	-	-	-	-	APECO1_2093; APECO1_2100
		ibeB	ACYG3R_17435	CA902_016270	KO11_20820	FVF73_02750; FVF73_17880	C_RS03045; c0658	APECO1_1476
		ibeC	ACYG3R_02835	CA902_021705	KO11_02555	FVF73_20530	C_RS23225; c4914	APECO1_2512
	Tia/Hek	*tia*	ACYG3R_05080; ACYG3R_05375; ACYG3R_21590	CA902_004250	-	-	-	APECO1_3530
Iron uptake	Aerobactin siderophore	*iucA*	-	-	-	-	C_RS10880; c2298	-

		iucB	-	-	-	-	C_RS10885; c2299	-
		iucC	-	-	-	-	C_RS10900; c2302	-
		iucD	-	-	-	-	C_RS10875; c2297	-
		iutA	-	-	-	-	C_RS10870; c2296	-
	Heme uptake	*chuA*	ACYG3R_01215	CA902_001290	-	-	C_RS05935; C_RS20360; c1265; c4308	APECO1_2948
		chuS	ACYG3R_01220	CA902_001295	-	-	C_RS20355; c4307	APECO1_2949
		chuT	ACYG3R_01210	CA902_001280	-	-	C_RS20370; c4313	APECO1_2947
		chuU	ACYG3R_01190	CA902_001260	-	-	C_RS20390; c4317	APECO1_2943
		chuW	ACYG3R_01205	CA902_001275	-	-	C_RS20375; c4314	APECO1_2946
		chuX	ACYG3R_01200	CA902_001270	-	-	C_RS20380; c4315	APECO1_2945
		chuY	ACYG3R_01195	CA902_001265	-	-	C_RS20385; c4316	APECO1_2944
	Iron-regulated element	*ireA*	ACYG3R_04890	-	-	-	C_RS24455; c5174	APECO1_3497
	Iron/manganese transport	*sitA*	-	-	-	-	c1600	APECO1_274
		sitB	-	-	-	-	C_RS07475; c1599	APECO1_273
		sitC	-	-	-	-	C_RS07470; c1598	APECO1_272
		sitD	-	-	-	-	C_RS07465; c1597	APECO1_271
	Salmochelin siderophore	*iroB*	-	-	-	-	C_RS05885; c1254	-
		iroC	-	-	-	-	C_RS05880; c1253	-
		iroD	-	-	-	-	C_RS05875; c1252	-
		iroE	-	-	-	-	C_RS05870; c1251	-

		iroN	-	-	-	-	C_RS03120; C_RS05865; c0669; c1250	APECO1_1465
	Yersiniabactin siderophore	*fyuA*	-	-	-	-	C_RS11540; c2436	APECO1_1063
		irp1	-	-	-	-	c2429	APECO1_1059
		irp2	-	-	-	-	c2424	APECO1_1058
		ybtA	-	-	-	-	C_RS11500; c2423	APECO1_1057
		ybtE	-	-	-	-	c2433	APECO1_1062
		ybtP	-	-	-	-	C_RS11495; c2422	APECO1_1056
		ybtQ	-	-	-	-	C_RS11490; c2421	APECO1_1055
		ybtS	-	-	-	-	C_RS11480; c2419	APECO1_1052
		ybtT	-	-	-	-	C_RS11530; c2432	APECO1_1061
		ybtU	-	-	-	-	C_RS11525; c2430	APECO1_1060
		ybtX	-	-	-	-	C_RS11485; c2420	APECO1_1054
	Cytochrome c maturation (*ccm*) locus (*Legionella*)	*ccmF*	-	-	-	-	C_RS12975; c2733	C_RS12975; c2733
	Ent siderophore (*Klebsiella*)	--	-	-	-	-	C_RS03160; c0678	C_RS03160; c0678
		--	-	-	-	-	C_RS03125; c0671	C_RS03125; c0671
	Enterobactin synthesis (*Shigella*)	*entA*	-	-	-	-	C_RS03185; c0683	C_RS03185; c0683
		entB	-	-	-	-	C_RS03180; c0682	C_RS03180; c0682
		entC	-	-	-	-	C_RS03170; c0680	C_RS03170; c0680
		entD	-	-	-	-	C_RS03110; c0668	C_RS03110; c0668
		entE	-	-	-	-	C_RS03175; c0681	C_RS03175; c0681
		entF	-	-	-	-	C_RS03135; c0673	C_RS03135; c0673

	Enterobactin transport (*Shigella*)	*fepB*	-	-	-	-	C_RS03165; c0679	C_RS03165; c0679
		fepC	-	-	-	-	C_RS03145; c0675	C_RS03145; c0675
		fepD	-	-	-	-	C_RS03155; c0677	C_RS03155; c0677
		fepG	-	-	-	-	C_RS03150; c0676	C_RS03150; c0676
	Heme biosynthesis (*Haemophilus*)	*hemC*	-	-	-	-	C_RS22315; c4724	C_RS22315; c4724
		hemE	-	-	-	-	C_RS23455; c4954	C_RS23455; c4954
		hemH	-	-	-	-	C_RS02735; c0595	C_RS02735; c0595
		hemL	-	-	-	-	C_RS00875; c0189	C_RS00875; c0189
		hemN	-	-	-	-	C_RS16815; C_RS22745; c3541; c4816	C_RS16815; C_RS22745; c3541; c4816
		shuV	-	-	-	-	C_RS20395; c4318	C_RS20395; c4318
LEE-encoded TTSS effectors	EspB	*espB*	-	-	-	-	-	-
	EspF	*espF*	-	-	-	-	-	-
	EspG	*espG*	-	-	-	-	-	-
	EspH	*espH*	-	-	-	-	-	-
	Mitochondria-associated protein Map	*map*	-	-	-	-	-	-
	SepZ/EspZ	*sepZ*	-	-	-	-	-	-
	Tir	*tir*	-	-	-	-	-	-
Non-LEE encoded	Cell-cycle-inhibitory factor Cif	*cif*	-	-	-	-	-	-
	EspFu/TccP	*espFu/tccP*	-	-	-	-	-	-

TTSS effectors	(Tir cytoskeleton coupling protein)							
	EspG2 (EPEC EspC island)	*espG2*	-	-	-	-	-	-
	EspJ	*espJ*	-	-	-	-	-	-
	EspK	*espK*	-	-	-	-	-	-
	EspL1	*espL1*	ACYG3R_11575	CA902_010435	-	-	-	-
	EspL2	*espL2*	-	-	-	-	-	-
	EspL4	*espL4*	-	-	KO11_02310	FVF73_20855	-	-
	EspM1	*espM1*	-	-	-	-	-	-
	EspM2	*espM2*	-	-	-	-	-	-
	EspN	*espN*	-	-	-	-	-	-
	EspO1-1	*espO1-1*	-	-	-	-	-	-
	EspO1-2	*espO1-2*	-	-	-	-	-	-
	EspR1	*espR1*	ACYG3R_12685	CA902_011520	-	-	-	-
	EspR3	*espR3*	-	-	-	-	-	-
	EspR4	*espR4*	-	-	-	-	-	-
	EspV	*espV*	-	-	-	-	-	-
	EspW	*espW*	-	-	-	-	-	-
	EspX1	*espX1*	ACYG3R_20490	CA902_019260	-	-	-	-
	EspX2	*espX2*	-	-	-	-	-	-
	EspX4	*espX4*	-	CA902_021140	KO11_02170	-	-	-
	EspX5	*espX5*	-	-	KO11_02035	FVF73_21175	-	-
	EspX6	*espX6*	ACYG3R_20950	CA902_019720	-	-	-	-

	EspX7	*espX7*	-	-	-	-	-	-
	EspY1	*espY1*	ACYG3R_20310	CA902_019080	-	-	-	-
	EspY2	*espY2*	ACYG3R_20245	CA902_019015	-	-	-	-
	EspY3	*espY3*	-	-	-	-	-	-
	EspY4	*espY4*	ACYG3R_23830	CA902_023210	-	-	-	-
	EspY5	*espY5*	-	-	-	-	-	-
	LifA/Efa1	*lifA/efa1*	-	-	-	-	-	-
	NleA	*nleA*	-	-	-	-	-	-
	NleB1	*nleB1*	-	-	-	-	-	-
	NleB2-1	*nleB2-1*	-	-	-	-	-	-
	NleB2-2	*nleB2-2*	-	-	-	-	-	-
	NleC	*nleC*	-	-	-	-	-	-
	NleD	*nleD*	-	-	-	-	-	-
	NleE-1	*nleE-1*	-	-	-	-	-	-
	NleE-2	*nleE-2*	-	-	-	-	-	-
	NleF	*nleF*	-	-	-	-	-	-
	NleG-1	*nleG-1*	-	-	-	-	-	-
	NleG-2	*nleG-2*	-	-	-	-	-	-
	NleG-3	*nleG-3*	-	-	-	-	-	-
	NleG2-2	*nleG2-2*	-	-	-	-	-	-
	NleG2-3	*nleG2-3*	-	-	-	-	-	-
	NleG2-4	*nleG2-4*	-	-	-	-	-	-
	NleG5-1	*nleG5-1*	-	-	-	-	-	-
	NleG5-2	*nleG5-2*	-	-	-	-	-	-

	NleG6-1	*nleG6-1*	-	-	-	-	-	-
	NleG6-2	*nleG6-2*	-	-	-	-	-	-
	NleG6-3	*nleG6-3*	-	-	-	-	-	-
	NleG7	*nleG7*	-	-	-	-	-	-
	NleG8-2	*nleG8-2*	-	-	-	-	-	-
	NleH1-1	*nleH1-1*	-	-	-	-	-	-
	NleH1-2	*nleH1-2*	-	-	-	-	-	-
	TccP2	*tccP2*	-	-	-	-	-	-
Regulation	AggR	*aggR*	-	-	-	-	-	-
	Carbon storage regulator A (*Legionella*)	*csrA*	-	-	-	-	C_RS15455; c3249	-
	GacS/GacA two-component system (Pseudomonas)	*gacA*	-	-	-	-	C_RS11025; c2327	-
	PhoPQ (*Salmonella*)	*phoP*	-	-	-	-	C_RS07040; c1509	-
		phoQ	-	-	-	-	C_RS07035; c1508	-
	RcsAB (*Klebsiella*)	--	-	-	-	-	C_RS13090; c2760	-
	Sigma A (*Mycobacterium*)	*rcsA*	-	-	-	-	C_RS11220; c2369	-
		sigA/rpoV	-	-	-	-	c3821	-
Secretion system	AAI/SCI-II T6SS	*aaiA*	-	-	-	-	-	-
		aaiB	-	-	-	-	-	-
		aaiC/hcp	-	-	-	-	-	-
		aaiD	-	-	-	-	-	-
		aaiE	-	-	-	-	-	-

		aaiF	-	-	-	-	-	-
		aaiH	-	-	-	-	-	-
		aaiI	-	-	-	-	-	-
		aaiJ	-	-	-	-	-	-
		aaiK	-	-	-	-	-	-
		aaiL	-	-	-	-	-	-
		aaiM	-	-	-	-	-	-
		aaiN	-	-	-	-	-	-
		clpV/aaiP	-	-	-	-	-	-
		icmF/aaiO	-	-	-	-	-	-
		vgrG	-	-	-	-	-	-
	ABC transporter for dispersin	*aatA*	-	-	-	-	-	-
		aatB	-	-	-	-	-	-
		aatC	-	-	-	-	-	-
		aatD	-	-	-	-	-	-
		aatP	-	-	-	-	-	-
	ACE T6SS	-	ACYG3R_19415	CA902_018190	KO11_01095	-	-	APECO1_1761
		-	-	-	-	-	-	APECO1_1753
		aec11	-	-	-	-	-	APECO1_1752
		aec14	-	-	-	-	-	APECO1_1755
		aec15	ACYG3R_12750; ACYG3R_17450	CA902_011590; CA902_016285	KO11_01130; KO11_20835	FVF73_07455	C_RS08835; C_RS08860; c1883; c1888	APECO1_1756; APECO1_596
		aec16	ACYG3R_19390	CA902_018165	KO11_01125	-	-	APECO1_1757

		aec17	ACYG3R_19395	CA902_018170	KO11_01115	-	-	APECO1_1758
		aec18	ACYG3R_19405	CA902_018180	KO11_01105	-	-	APECO1_1759
		aec19	ACYG3R_19410	CA902_018185	KO11_01100	-	-	APECO1_1760
		aec22	ACYG3R_19420	CA902_018195	KO11_01090	-	-	APECO1_1762
		aec23	ACYG3R_19425	CA902_018200	KO11_01085	-	-	APECO1_1763
		aec24	ACYG3R_19430	CA902_018205	KO11_01080	-	-	APECO1_1764
		aec25	ACYG3R_19435	CA902_018210	KO11_01075	-	-	APECO1_1765
		aec26	-	-	KO11_01070	-	-	APECO1_1766
		aec27/clpV	ACYG3R_19445	CA902_018220	KO11_01065	-	-	APECO1_1767
		aec28	ACYG3R_19450	CA902_018225	KO11_01060	-	-	APECO1_1768
		aec29	ACYG3R_19455	CA902_018230	KO11_01055	-	-	APECO1_1769
		aec30	ACYG3R_19460	CA902_018235	KO11_01050	-	-	APECO1_1770
		aec31	ACYG3R_19465	CA902_018240	KO11_01045	FVF73_01325	-	APECO1_1771
		aec32	ACYG3R_19470	CA902_018245	KO11_01040	FVF73_01320	-	APECO1_1772
		aec7	-	-	-	-	-	APECO1_1749
		aec8	-	-	-	-	-	APECO1_1750
	LEE locus encoded TTSS	-	-	-	-	-	-	-
		-	-	-	-	-	-	-
		-	-	-	-	-	-	-
		-	-	-	-	-	-	-
		-	-	-	-	-	-	-
		cesD2	-	-	-	-	-	-
		cesD	-	-	-	-	-	-
		cesF	-	-	-	-	-	-

		cesT	-	-	-	-	-	-
		escC	-	-	-	-	-	-
		escD	-	-	-	-	-	-
		escF	-	-	-	-	-	-
		escI	-	-	-	-	-	-
		escJ	-	-	-	-	-	-
		escK	-	-	-	-	-	-
		escL	-	-	-	-	-	-
		escN	-	-	-	-	-	-
		escO	-	-	-	-	-	-
		escP	-	-	-	-	-	-
		escR	-	-	-	-	-	-
		escS	-	-	-	-	-	-
		escT	-	-	-	-	-	-
		escU	-	-	-	-	-	-
		escV	-	-	-	-	-	APECO1_928
		espA	-	-	-	-	-	-
		espB	-	-	-	-	-	-
		espD	-	-	-	-	-	-
		etgA	-	-	-	-	-	-
		glrA	-	-	-	-	-	-
		glrR	-	-	-	-	-	-
		ler	-	-	-	-	-	-
		sepD	-	-	-	-	-	-

		sepL	-	-	-	-	-	-
		sepQ	-	-	-	-	-	-
	SCI-I T6SS	-	-	-	KO11_08885	-	C_RS16065; c3385	APECO1_3718
		-	-	-	KO11_08880	-	C_RS16070; c3386	APECO1_3717
		-	-	-	KO11_08875	-	C_RS16075; c3387	APECO1_3716
		-	-	-	KO11_08870	-	C_RS16080; c3388	APECO1_3715
		-	-	-	KO11_08865	-	C_RS16085; c3389	APECO1_3714
		-	-	-	KO11_08860	-	C_RS16090; c3391	APECO1_3713
		-	-	-	KO11_08855	-	C_RS16095; c3392	APECO1_3712
		-	-	-	-	-	-	-
		-	-	-	-	-	-	-
		-	-	-	KO11_08835; KO11_08850	-	C_RS16100; c3393	APECO1_3711
		-	-	-	-	-	-	-
		-	-	-	-	-	-	-
		-	-	-	-	-	-	-
		-	-	-	-	-	C_RS16115; c3396	-
		-	-	-	-	-	-	-
		-	-	-	KO11_08815	-	C_RS16125; c3398	APECO1_3705
		-	-	-	KO11_08790; KO11_08810	-	C_RS16130; c3399	APECO1_3702
		-	-	-	-	-	-	-
		-	-	-	KO11_08750	-	c3400	APECO1_3698
		-	-	-	KO11_08745	-	C_RS16140	APECO1_3697

		-	-	-	KO11_08740	-	C_RS16145; c3401	APECO1_3696
		-	-	-	KO11_08735	-	C_RS16150; c3402	APECO1_3695
		-	-	-	-	-	-	-
		-	-	-	-	-	-	-
		-	-	-	-	-	-	-
		-	-	-	-	-	-	-
		-	-	-	-	-	C_RS16155; c3403	APECO1_3694
	EPS type II secretion system (*Vibrio*)	*epsE*	-	-	-	-	C_RS19405; c4097	-
	Flagella (cluster I) (*Yersinia*)	*flgB*	-	-	-	-	C_RS06295; c1342	-
	Flagella (cluster I) (*Yersinia*)	*flgC*	-	-	-	-	C_RS06300; c1343	-
		flgD	-	-	-	-	C_RS06305; c1344	-
		flgE	-	-	-	-	C_RS06310; c1345	-
		flgF	-	-	-	-	C_RS06315; c1346	-
		flgG	-	-	-	-	C_RS06320; c1347	-
		flgH	-	-	-	-	C_RS06325; c1348	-
		flgI	-	-	-	-	C_RS06330; c1349	-
		flgJ	-	-	-	-	C_RS06335; c1350	-
		flgK	-	-	-	-	C_RS06340; c1351	-
		flgL	-	-	-	-	C_RS06345; c1352	-
		flgM	-	-	-	-	C_RS06285; c1340	-
		flhA	-	-	-	-	C_RS10860; c2293	-
		flhB	-	-	-	-	C_RS10865; c2294	-

		flhC	-	-	-	-	C_RS10920; c2306	-
		flhD	-	-	-	-	C_RS10925; c2308	-
		fliA	-	-	-	-	C_RS11075; c2337	-
		fliE	-	-	-	-	C_RS11150; c2353	-
		fliF	-	-	-	-	C_RS11155; c2354	-
		fliG	-	-	-	-	C_RS11160; c2355	-
		fliH	-	-	-	-	C_RS11165; c2357	-
		fliI	-	-	-	-	C_RS11170; c2358	-
		fliJ	-	-	-	-	C_RS11175; c2359	-
		fliL	-	-	-	-	C_RS11185; c2361	-
		fliM	-	-	-	-	C_RS11190; c2362	-
		fliN	-	-	-	-	C_RS11195; c2363	-
		fliP	-	-	-	-	C_RS11205; c2365	-
		fliQ	-	-	-	-	C_RS11210; c2366	-
		fliR	-	-	-	-	C_RS11215; c2367	-
		fliS	-	-	-	-	C_RS11090; c2340	-
		fliZ	-	-	-	-	C_RS11070; c2336	-
	Mxi-Spa TTSS effectors controlled by MxiE (*Shigella*)	*ipaH*	-	-	-	-	C_RS15255; c3208	-
	T2SS (Type II secretion system) (*Shigella*)	*gspM*	-	-	-	-	C_RS17540; c3699	-
	T2SS (*Aeromonas*)	*exeD*	-	-	-	-	C_RS19400; c4096	-
		exeG	-	-	-	-	C_RS19415; c4099	-

	T4SS effectors (*Coxiella*)	--	-	-	-	-	C_RS18625; c3927	-
		--	-	-	-	-	C_RS10790; c2278	-
	T6SS-II (*Klebsiella*)	*clpB*	-	-	-	-	C_RS14805; c3114	-
	T6SS (*Aeromonas*)	--	-	-	-	-	C_RS08830; C_RS08855; c1882; c1887	-
	Flagella (cluster I) (*Yersinia*)	*fliC*	ACYG3R_10485	CA902_009355	-	-	-	-
Toxin	α-hemolysin	*hlyA*	-	-	-	-	C_RS16950; c3570	-
		hlyB	-	-	-	-	C_RS16955; c3573	-
		hlyC	-	-	-	-	C_RS16945; c3569	-
		hlyD	-	-	-	-	C_RS16960; c3574	-
	Colicin-like Usp	*usp*	-	-	-	-	C_RS00625	APECO1_1876
	Cytolethal distending toxin	*cdtA*	-	-	-	-	-	APECO1_542
		cdtB	-	-	-	-	-	APECO1_543
		cdtC	-	-	-	-	-	APECO1_544
	Cytotoxic necrotizing factor 1	*cnf1*	-	-	-	-	-	-
	Enterotoxin 1	*set1A*	-	-	-	-	-	-
		set1B	-	-	-	-	-	-
	Enterotoxin SenB/TieB	*senB*	-	-	-	-	-	-
	Heat-labile enterotoxin	*eltA*	-	-	-	-	-	-
		eltB	-	-	-	-	-	-

	Heat-stable enterotoxin 1 (EAST1)	*astA*	-	-	-	-	-	-
	Hemolysin/cytolysin A	*hlyE/clyA*	ACYG3R_14425	CA902_013270	KO11_16920	-	c1630	-
	Shiga-like toxin	*stx1A*	-	-	-	-	-	-
		stx1B	-	-	-	-		-
		stx2A	-	-	-	-		-
		stx2B	-	-	-	-	-	-
	Colibactin (*Klebsiella*)	*clbA*	-	-	-	-	C_RS11715; c2471	-
		clbC	-	-	-	-	C_RS11700; c2468	-
		clbD	-	-	-	-	C_RS11695; c2467	-
		clbE	-	-	-	-	C_RS11690; c2466	-
		clbF	-	-	-	-	C_RS11685; c2464	-
		clbG	-	-	-	-	C_RS11680; c2463	-
		clbH	-	-	-	-	C_RS11675; c2461	-
		clbI	-	-	-	-	C_RS11670; c2460	-
		clbJ	-	-	-	-	C_RS11665; c2459	-
		clbK	-	-	-	-	C_RS11660; c2458	-
		clbL	-	-	-	-	C_RS11655; c2457	-
		clbM	-	-	-	-	C_RS11650; c2456	-
		clbN	-	-	-	-	c2455	-
		clbO	-	-	-	-	C_RS11640; c2453	-
		clbP	-	-	-	-	C_RS11635; c2452	-
		clbQ	-	-	-	-	C_RS11630; c2451	-

	Heat-stable cytotonic enterotoxin	*ast*	-	-	-	-	C_RS05520; c1174	-
	Hemolysin HlyA (*Aeromonas*)	*hlyA*	-	-	-	-	C_RS25105; c5316	-
	Hemolysin III (*Aeromonas*)	--	-	-	-	-	C_RS16530; c3480	-
	Phytotoxin phaseolotoxin (*Pseudomonas*)	*cysC1*	-	-	-	-	C_RS15755; c3317	-
Adherence and invasion	EF-Tu (*Francisella*)	--	-	-	-	-	C_RS19470; C_RS23370; c4111; c4935	-
Amino acid and purine metabolism	Glutamine synthesis (*Mycobacterium*)	*glnA1*	-	-	-	-	C_RS22765; c4819	-
Anaerobic respiration	Nitrate reductase (*Mycobacterium*)	*narH*	-	-	-	-	C_RS07875; C_RS08920; c1686; c1899	-
Antiphagocy tosis	Alginate regulation (*Pseudomonas*)	*algU*	-	-	-	-	C_RS14700; c3097	-
	Capsular polysaccharide (*Vibrio*)	*rmlB*	-	-	-	-	C_RS22225; c4708	-
		wbjD/wecB	-	-	-	-	C_RS22215; c4706	-
		wecC	-	-	-	-	C_RS22220; c4707	-
	Capsule (*Klebsiella*)	--	-	-	-	-	C_RS12125; C_RS12130; C_RS12205;	-

							C_RS12230; C_RS12235; c2555; c2556; c2572; c2578; c2579	
		galF	-	-	-	-	C_RS12180; c2567	-
		wcaI	-	-	-	-	C_RS12220; c2576	-
		wzc	-	-	-	-	C_RS12270; c2586	-
Biofilm formation	PNAG (Polysaccharide poly-N-acetylglucosamine) (*Acinetobacter*)	*pgaC*	-	-	-	-	C_RS05455; c1161	-
Cell surface components	Trehalose-recycling ABC transporter (*Mycobacterium*)	*sugC*	-	-	-	-	C_RS08385; C_RS23710; c1790; c5005	-
Colonization and Immune evasion	Capsule biosynthesis and transport (*Campylobacter*)	*kpsT*	-	-	-	-	C_RS17530; c3697	-
Efflux pump	AcrAB (*Klebsiella*)	--	-	-	-	-	C_RS02670; C_RS08255; C_RS19105; c0581; c1761; c4031; c4324	-
		acrA	-	-	-	-	C_RS20430	-

		acrB	-	-	-	-	C_RS02665; C_RS08260; C_RS14230; C_RS19110; C_RS20435; c0580; c1764; c2997; c4032; c4325	-
	FarAB (*Neisseria*)	*farB*	-	-	-	-	C_RS13765; C_RS15390; c2902; c3239	-
Endotoxin	LOS (*Haemophilus*)	*gmhA/lpcA*	-	-	-	-	C_RS01710; c0372	-
		htrB	-	-	-	-	C_RS06200; c1320	-
		kdsA	-	-	-	-	C_RS07815; c1674	-
		kdtA	-	-	-	-	C_RS21035; c4457	-
		kpsF	-	-	-	-	C_RS17475; c3686	-
		lpxA	-	-	-	-	C_RS01005; c0218	-
		lpxB	-	-	-	-	C_RS01010; c0219	-
		lpxC	-	-	-	-	C_RS00530; c0114	-
		lpxD	-	-	-	-	C_RS00995; c0216	-
		msbA	-	-	-	-	C_RS04965; c1054	-
		opsX/rfaC	-	-	-	-	C_RS20985; c4447	-
		orfM	-	-	-	-	C_RS16810; c3540	-
		rfaD	-	-	-	-	C_RS20975; c4445	-

		rfaE	-	-	-	-	C_RS18065; c3800	-
		rfaF	-	-	-	-	C_RS20980; c4446	-
		wecA	-	-	-	-	C_RS22205; c4704	-
Enzyme	*Streptococcal* enolase (*Streptococcus*)	*eno*	-	-	-	-	C_RS15880; c3344	-
Fimbrial adherence determinants	Sta (*Salmonella*)	*staB*	-	-	-	-	C_RS00805; c0171	-
		staC	-	-	-	-	C_RS00800; c0170	-
	Stc (*Salmonella*)	*stcB*	-	-	-	-	C_RS12510; c2637	-
		stcC	-	-	-	-	C_RS12505; c2636	-
	Stf (*Salmonella*)	*stcD*	-	-	-	-	C_RS12500; c2635	-
		stfC	-	-	-	-	C_RS13675; c2883	-
		stfD	-	-	-	-	C_RS13670; c2882	-
		stfE	-	-	-	-	C_RS13665; c2881	-
		stfF	-	-	-	-	C_RS13660; c2880	-
Immune evasion	Capsule (*Acinetobacter*)	-	ACYG3R_09930; ACYG3R_09935	-	-	-	-	-
	Capsule (*Klebsiella*)	-	-	-	-	FVF73_10500; FVF73_10510; FVF73_10520; FVF73_10525	-	APECO1_1128
		wzc	-	-	-	FVF73_10505	-	-
	Capsule (*Streptococcus*)	*rmlA*	-	-	-	-	-	APECO1_1129

	Exopolysaccharide (*Haemophilus*)	*galE*	-	CA902_004015	-	-	C_RS03955; C_RS12150; c0835; c2560	-
		galU	-	-	-	-	C_RS07955; c1700	-
		mrsA/glmM	-	-	-	-	C_RS18655; c3932	-
		pgi	-	-	-	-	C_RS23650; c4991	-
	LPS (*Brucella*)	*acpXL*	-	-	-	-	C_RS06400; c1364	-
Lipid and fatty acid metabolism	Isocitrate lyase (*Mycobacterium*)	*icl*	-	-	-	-	C_RS23555; c4972	-
	Pantothenate synthesis (*Mycobacterium*)	*panD*	-	-	-	-	C_RS00755; c0160	-
Magnesium uptake	Mg^{2+} transport (*Salmonella*)	*mgtB*	-	-	-	-	C_RS25220; c5341	-
Motility	Flagella (*Bordetella*)	*motB*	-	-	-	-	C_RS10910; c2304	-
Nonfimbrial adherence determinants	SinH (*Salmonella*)	*sinH*	-	-	-	-	C_RS14400; c3031	-
Nutritional factor	Allantoin utilization (*Klebsiella*)	--	-	-	-	-	C_RS02855; c0619	-
		--	-	-	-	-	C_RS02860; c0620	-
		--	-	-	-	-	C_RS02865; c0621	-
		--	-	-	-	-	C_RS02890; c0626	-
		--	-	-	-	-	C_RS02910; c0630	-
		--	-	-	-	-	C_RS02915; c0631	-

Nutritional virulence	Biotin metabolism (*Francisella*)	*bioB*	-	-	-	-	C_RS04045; c0855	-
	Cysteine acquisition (*Francisella*)	--	-	-	-	-	C_RS20045; c4236	-
	Pyrimidine biosynthesis (*Francisella*)	--	-	-	-	-	C_RS00190; c0041	-
Others	MsbB2 (*Shigella*)	*msbB2*	-	-	-	-	C_RS10745; c2269	-
	O-antigen (*Yersinia*)	--	-	-	-	-	C_RS12135; C_RS12140; C_RS12210; C_RS12215; C_RS13525; C_RS22210; c2557; c2558; c2573; c2575; c2850; c4705	-
Protease	IcsP (SopA) (*Shigella*)	*icsP/sopA*	-	-	-	-	C_RS03015; c0652	-
Quorum sensing	Autoinducer-2 (*Vibrio*)	*luxS*	-	-	-	-	C_RS15405; c3244	-
Serum resistance and immune evasion	Capsule (*Francisella*)	--	-	-	-	-	C_RS19685; c4156	-
	LPS (*Francisella*)	--	-	-	-	-	C_RS21950; c4655	-

Stress adaptation	Catalase-peroxidase (*Mycobacterium*)	*katG*	-	-	-	-	C_RS23160; c4900	-
	SodCI (*Salmonella*)	*sodCI*	-	-	-	-	C_RS09630; c2038	-
Serum resistance	LPS rfb locus (*Klebsiella*)	-	-	-	-	FVF73_10440; FVF73_10445; FVF73_18905	-	-
		rmlA					C_RS22230; c4709	-
		rmlD	-	-	-	-	-	APECO1_1130
Fimbrial adherence determinants	Lpf (*Salmonella*)	*lpfB*	-	-	KO11_05000	-	-	-
		lpfC	-	-	KO11_05005	-	-	-
		lpfE	-	-	KO11_05015	-	-	-

Table S6. Comparison of virulence factors in pEcE.ESBL.COL-01 and pCFSAN061771_01

VF class	Virulence factors	Related genes	pEcE.ESBL.COL-01	pCFSAN061771_01
			JBTWAA010000002	CP042897
	Vacuolating autotransporter gene	*vat*	ACYG3R_24150	CA902_023610
Iron uptake	Aerobactin siderophore	*iucA*	ACYG3R_24715	CA902_023970
		iucB	ACYG3R_24710	CA902_023975
		iucC	ACYG3R_24705	CA902_023980
		iucD	ACYG3R_24700	CA902_023985
		iutA	ACYG3R_24695	CA902_023990
	Iron/manganese transport	*sitA*	ACYG3R_24755	CA902_023925
		sitB	ACYG3R_24750	CA902_023930
		sitC	ACYG3R_24745	CA902_023935
		sitD	ACYG3R_24740	CA902_023940
	Salmochelin siderophore	*iroB*	ACYG3R_24030	CA902_023740
		iroC	ACYG3R_24035	CA902_023735
		iroD	ACYG3R_24040	CA902_023730
		iroE	ACYG3R_24045	CA902_023725
		iroN	ACYG3R_24050	CA902_023720

Table S7. Comparative analysis of polymyxin resistance-associated lipopolysaccharide (LPS) modification enzymes across five strains

**Enzyme (Locus tag)**	**Strain (Genome accession No., Locus tag)**	**Identity%, Coverage%**
ArnF (ACYG3R_08775) 4-amino-4-deoxy-L-arabinose-phosphoundecaprenol flippase	*Escherichia coli* strain CFSAN061771 (CP042896, CA902_007660)	100, 100
	Escherichia coli strain KO11FL (CP002970, KO11_11390)	98, 100
	Escherichia coli strain 042 (CP042934, FVF73_11520)	98, 100
	Escherichia coli strain CFT073 (CP051263, c2801)	97,100
	Escherichia coli strain APEC O1 (CP000468, APECO1_4303)	96, 100
ArnE (ACYG3R_08780) 4-amino-4-deoxy-L-arabinose-phosphoundecaprenol flippase	*Escherichia coli* strain CFSAN061771 (CP042896, CA902_007665)	100, 100
	Escherichia coli strain KO11FL (CP002970, KO11_11395)	100, 100
	Escherichia coli strain 042 (CP042934, FVF73_11515)	100, 100
	Escherichia coli strain CFT073 (NC_004431, c2800)	96,100
	Escherichia coli strain APEC O1 (CP000468)	-
ArnT (ACYG3R_08785) lipid IV (A) 4-amino-4-deoxy-L-arabinosyltransferase	*Escherichia coli* strain CFSAN061771 (CP042896, CA902_007670)	100, 100
	Escherichia coli strain KO11FL (CP002970, KO11_11400)	98, 100
	Escherichia coli strain 042 (CP042934, FVF73_11510)	98, 100
	Escherichia coli strain CFT073 (NC_004431, c2799)	99,100
	Escherichia coli strain APEC O1 (CP000468, APECO1_4304)	99, 100
ArnD (ACYG3R_08790)	*Escherichia coli* strain CFSAN061771	100, 100

4-deoxy-4-formamido-L-arabinose-phosphoundecaprenol deformylase	(CP042896, CA902_007675)	
	Escherichia coli strain KO11FL (CP002970, KO11_11405)	97, 100
	Escherichia coli strain 042 (CP042934, FVF73_11505)	96, 100
	Escherichia coli strain CFT073 (NC_004431, c2798)	97, 99
	Escherichia coli strain APEC O1 (CP000468, APECO1_4305)	97, 99
ArnA (ACYG3R_08795) bifunctional UDP-4-amino-4-deoxy-L-arabinose formyltransferase	*Escherichia coli* strain CFSAN061771 (CP042896, CA902_007680)	100, 100
	Escherichia coli strain KO11FL (CP002970, KO11_11410)	96, 100
	Escherichia coli strain 042 (CP042934, FVF73_11500)	96, 100
	Escherichia coli strain CFT073 (NC_004431, c2797)	97,100
	Escherichia coli strain APEC O1 (CP000468, APECO1_4306)	97, 100
ArnC (ACYG3R_08800) Undecaprenyl-phosphate 4-deoxy-4-formamido-L-arabinose transferase	*Escherichia coli* strain CFSAN061771 (CP042896, CA902_007685)	100, 100
	Escherichia coli strain KO11FL (CP002970, KO11_11415)	99, 100
	Escherichia coli strain 042 (CP042934, FVF73_11495)	98, 100
	Escherichia coli strain CFT073 (NC_004431, c2796)	99,100
	Escherichia coli strain APEC O1 (CP000468, APECO1_4307)	99, 100
ArnB (ACYG3R_08805) UDP-4-amino-4-deoxy-L-arabinose aminotransferase	*Escherichia coli* strain CFSAN061771 (CP042896, CA902_007690)	100, 98
	Escherichia coli strain KO11FL (CP002970, KO11_11420)	99, 100
	Escherichia coli strain 042	99, 98

	(CP042934, FVF73_11490)	
	Escherichia coli strain CFT073 (NC_004431, c2795)	98, 98
	Escherichia coli strain APEC O1 (CP000468, APECO1_4308)	97, 100

Table S8. Viable counts of donor strains (ECE.ESBL.COL), transconjugants (25DN-Amp), and the calculated conjugation transfer frequencies

Replicate	Donor (CFU/mL)	Transconjugants (CFU/mL)	Transconjugantion frequency (Transconjugants/Donor)
1	1.66×10^{9}	1.8×10^{4}	1.08×10^{-5}
2	1.3×10^{9}	1.2×10^{4}	9.23×10^{-6}
3	1.38×10^{9}	2.8×10^{4}	2.03×10^{-5}
